# Cyclophilin D regulates the dynamic assembly of mitochondrial ATP synthase into synthasomes

**DOI:** 10.1038/s41598-017-14795-x

**Published:** 2017-11-03

**Authors:** Gisela Beutner, Ryan E. Alanzalon, George A. Porter

**Affiliations:** 1 Department of Pediatrics (Cardiology), University of Rochester, Rochester, New York, 14642 United States; 2 Department of Pharmacology and Physiology, University of Rochester, Rochester, New York, 14642 United States; 3 Department of Medicine (Aab Cardiovascular Research Institute), University of Rochester, Rochester, New York, 14642 United States

## Abstract

Mitochondrial electron transport is essential for oxidative phosphorylation (OXPHOS). Electron transport chain (ETC) activity generates an electrochemical gradient that is used by the ATP synthase to make ATP. ATP synthase is organized into supramolecular units called synthasomes that increase the efficiency of ATP production, while within ATP synthase is the cyclophilin D (CypD) regulated mitochondrial permeability transition pore (PTP). We investigated whether synthasomes are dynamic structures that respond to metabolic demands and whether CypD regulates this dynamic. Isolated heart mitochondria from wild-type (WT) and CypD knockout (KO) mice were treated to either stimulate OXPHOS or open the PTP. The presence and dynamics of mitochondrial synthasomes were investigated by native electrophoresis, immunoprecipitation, and sucrose density centrifugation. We show that stimulation of OXPHOS, inhibition of the PTP, or deletion of CypD increased high order synthasome assembly. In contrast, OXPHOS inhibition or PTP opening increased synthasome disassembly in WT, but not in CypD KO heart mitochondria. CypD activity also correlated with synthasome assembly in other tissues, such as liver and brain. We conclude that CypD not only regulates the PTP, but also regulates the dynamics of synthasome assembly depending on the bioenergetic state of the mitochondria.

## Introduction

The primary function of mitochondria is to generate ATP by OXPHOS. For this process, electrons from NADH and FADH_2_ enter the ETC, which consists of four multi-subunit protein complexes (Cx-I to Cx-IV) that are encoded by both mitochondrial and nuclear genes^1^. During ETC activity, Cx-I, -III and -IV pump protons from the mitochondrial matrix into the intermembrane space. The resulting proton and electrical gradient provides the driving force for ATP synthase (also known as Cx-V) to generate ATP^2^.

The protein complexes of the ETC and the ATP synthase assemble further into two types of supercomplexes that may increase overall efficiency of OXPHOS^3^: respirasomes (Cx-I, ubiquinone, Cx-III, cytochrome *c*, and Cx-IV)^4^ and synthasomes (ATP synthase, adenine nucleotide translocase (ANT), phosphate carrier (PiC)^5^, and in striated myocytes the mitochondrial creatine kinase (mtCK))^5–7^. For example, respirasomes may create micro-compartments for proton and electron transfer^8–11^. In addition, higher order assemblies of synthasomes may mold the cristae into tubular structures and create micro-compartments that increase the efficiency of ATP synthesis^12–16^ and the transfer of energy from the mitochondria to cytoplasmic energy consuming metabolic pathways^7^. Three models are proposed to explain ETC supercomplex assembly and function^8,11^. In the solid state model, the ETC is functional only as respirasomes and synthasomes. In the fluid model, the complexes are independent entities embedded in the inner membrane, with ubiquinone and cytochrome *c* acting as mobile electron carriers. The plasticity model suggests that the ETC is a dynamic structure that varies between solid and fluid states with supercomplex assembly dependent on metabolic demands.

It is unclear whether these models can be applied to synthasomes. Furthermore, the assembly of ATP synthase into dimers, tetramers, oligomers and synthasomes is poorly understood. Recent data shows that subunits Al6, e, f, g and b of the F_o_ subunit of the ATP synthase are important for dimerization^13,15^, but the mechanisms that control higher order assembly remain unclear.

We have shown that respirasome assembly is developmentally regulated^17^. In the embryonic heart, respirasome assembly begins after embryonic day 11.5 and coincides developmentally with the activation of the ETC and closing of the PTP^17,18^. Although the subject is controversial, we and others have shown that the PTP is a large channel within the ATP synthase^19–25^, and its opening de-energizes mitochondria and uncouples electron transport from ATP production. Opening and closing of the PTP regulates physiological and pathological pathways^26,27^. We recently proposed that a relative or absolute disassembly of the ATP synthase leads to exposure of its membranous c-subunit ring, which creates the pore of the PTP^19^, adding therefore another layer to synthasome assembly and disassembly that is poorly understood. The PTP is regulated by CypD, a peptidyl-prolyl *cis*/*trans* isomerase (PPIase) in the mitochondrial matrix. Deletion of CypD or inhibiting it with cyclosporin A (CsA) desensitizes mitochondria against PTP opening, but how CypD does this, remains a mystery^28–30^.

We hypothesized that PTP opening is linked to supercomplex assembly and that the bioenergetic state of the cell may control a dynamic formation of synthasomes in the adult heart. In WT mouse hearts, we found that opening of the PTP led to disassembly of synthasomes, while stimulation of OXPHOS and the deletion or inhibition of CypD preserved synthasomes. Furthermore, the levels of CypD containing synthasomes varied amongst different tissues, but corresponded to the PPIase activity of CypD. Our data suggest a novel mechanism that links synthasome assembly and PTP formation: the primary function of CypD is to maintain the dynamics of synthasomes, and its ability to regulate the PTP may be secondary to synthasome assembly.

## Results

### Synthasomes are present in cardiac mitochondria

Purification of the mitochondrial synthasome, a protein complex consisting of ATP synthase, ANT and PiC has been described^5^. It has also been hypothesized that mtCK is part of this complex in striated muscle to mediate the transport of energy equivalents through a network of creatine kinases into the cytosol^6^. Using mild experimental conditions, we sought to establish the presence of synthasomes in hearts from adult C57BL/6 N mice and to determine whether the formation of synthasomes is a dynamic process that depends on bioenergetic demands.

**Figure 1:**
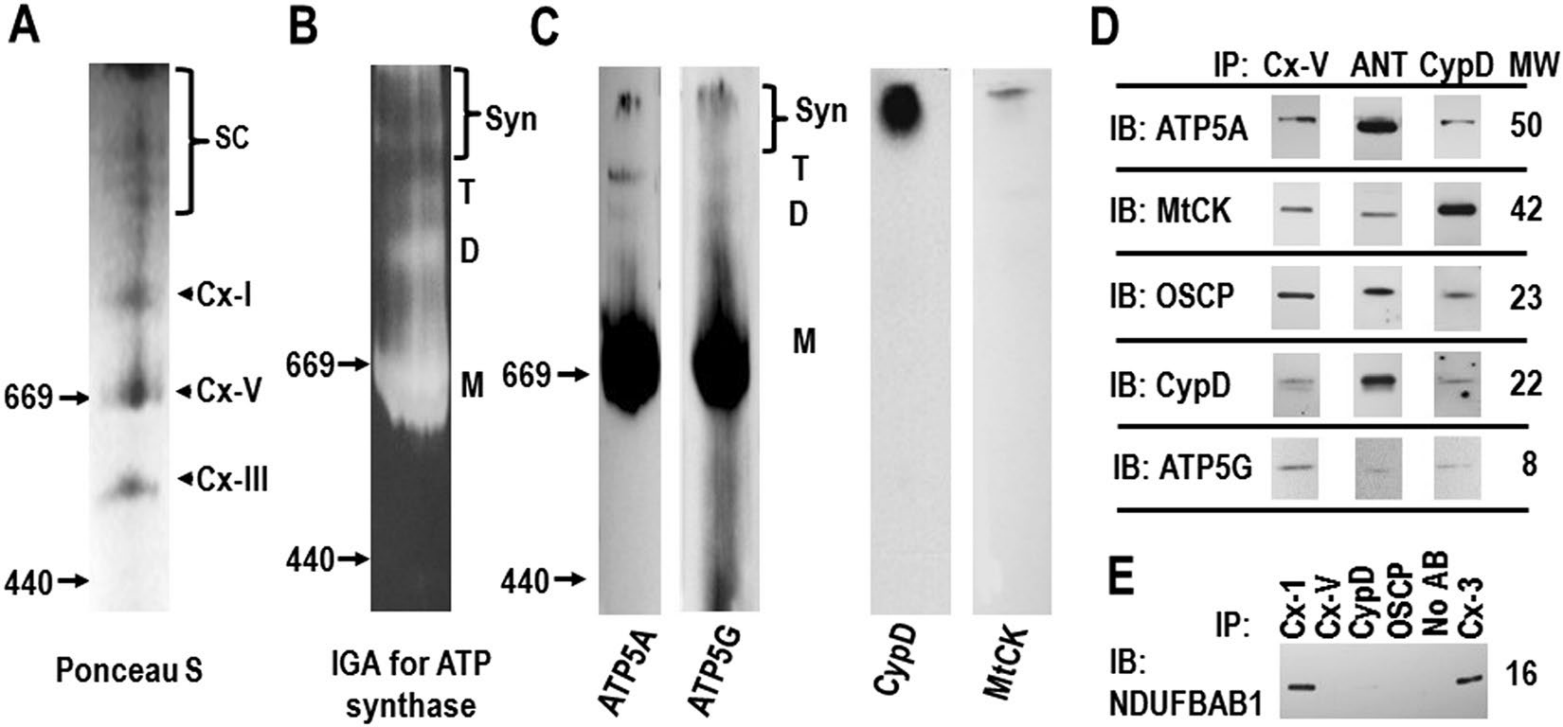
Synthasomes in mitochondria from mouse hearts. (**A**) Ponceau S staining of a CN PAGE after transfer onto nitrocellulose membranes shows a distinct pattern of monomeric ETC complexes (Cx) I, V, and III and supercomplexes (SC). (**B**) Representative in-gel-assay (IGA; n = 8) shows ATP hydrolyzing activity in multiple bands. (**C**) Immunoblotting (IB) for ATP5A (n ≥ 10) and ATP5G (n = 2) demonstrate monomers (M), dimers (D), tetramers (T) and synthasomes (Syn) in CN gels. In parallel labeling, CypD (n = 5) and mtCK (n = 5) are present only in high molecular weight protein complexes. (**D**) Immunoprecipitation (IP) of the synthasome with antibodies against ATP synthase (Cx-V, n ≥ 5), ANT (n = 3) and CypD (n = 2) followed by IB against ATP5A (n = 5), mtCK (n = 3), OSCP (n = 2), CypD (n = 3) and ATP5G (n = 1). (**E**) Precipitates obtained with antibodies against ATP synthase (Cx-V), OSCP, and CypD do not contain the subunit NDUFAB1 of Cx-I, while IP of Cx-I and Cx-III do (n = 3). Positions of the molecular weight (MW) markers (in kDa) are indicated by arrows (**A**–**C**).

Clear native (CN) electrophoresis allows separation and resolution of very high molecular weight, functional protein complexes and was used to examine the presence of synthasomes (Fig. 1A,B,C; entire lanes of these blots are shown in Supplementary (S) Fig. S1A, S1B and S1C). Ponceau S labeling after CN electrophoresis demonstrated several protein complexes (supercomplexes) with a molecular weight (MW) higher than the monomers of ATP synthase (≈660 kDa) and Cx-I (880 kDa) in heart mitochondria from adult WT mice (Fig. 1A, Fig. S1A). These supercomplexes were most reliably preserved by using a lauryl maltoside:protein ratio of 2 µg:1 µg (w/w) to solubilize mitochondrial membranes. Repeated cycles of freezing and thawing destroyed them. In-gel assays (IGA) for the ATP hydrolyzing activity of the ATP synthase and immunoblotting (IB) for the α-subunit (ATP5A) and the c-subunit (ATP5G) of the ATP synthase identified at least 4 ATP synthase-containing protein complexes, consistent with the size of the monomer (M; ≈660 kD), dimer (D; ≈1,300 kD), tetramer (T; ≈2,000 kD) and higher molecular weight synthasomes (Syn; Fig. 1C and Fig. S1C). IB after CN electrophoresis demonstrated that CypD and mtCK co-migrate only with the oligomeric supercomplexes (Figs. 1C, S1C).

Immunoprecipitating ATP synthase from freshly isolated mitochondria with an antibody that recognizes its catalytic (F_1_) portion precipitated the ATP synthase subunits ATP5A, OSCP (oligomycin sensitivity conferring protein) and ATP5G, as well as CypD and mtCK (Figs. 1D, S2 A–C). Conversely, antibodies to ANT and CypD co-precipitated ATP5A, ATP5G, OSCP and mtCK (Figs. 1D, S2 A–C), further supporting the idea that these proteins form functional synthasomes. The ATP synthase, OSCP and CypD antibodies did not precipitate the Cx-I protein NDUFAB1 (Figs. 1E, S2D), while Cx-I and Cx-III antibodies precipitated NDUFAB1 (Figs. 1E, S2D). Based on the co-localization and co-precipitation of ATP5A, mtCK and CypD in the largest protein complexes entering the native gel, we define these protein complexes as synthasomes.

### Respiratory state controls synthasome assembly

**Figure 2:**
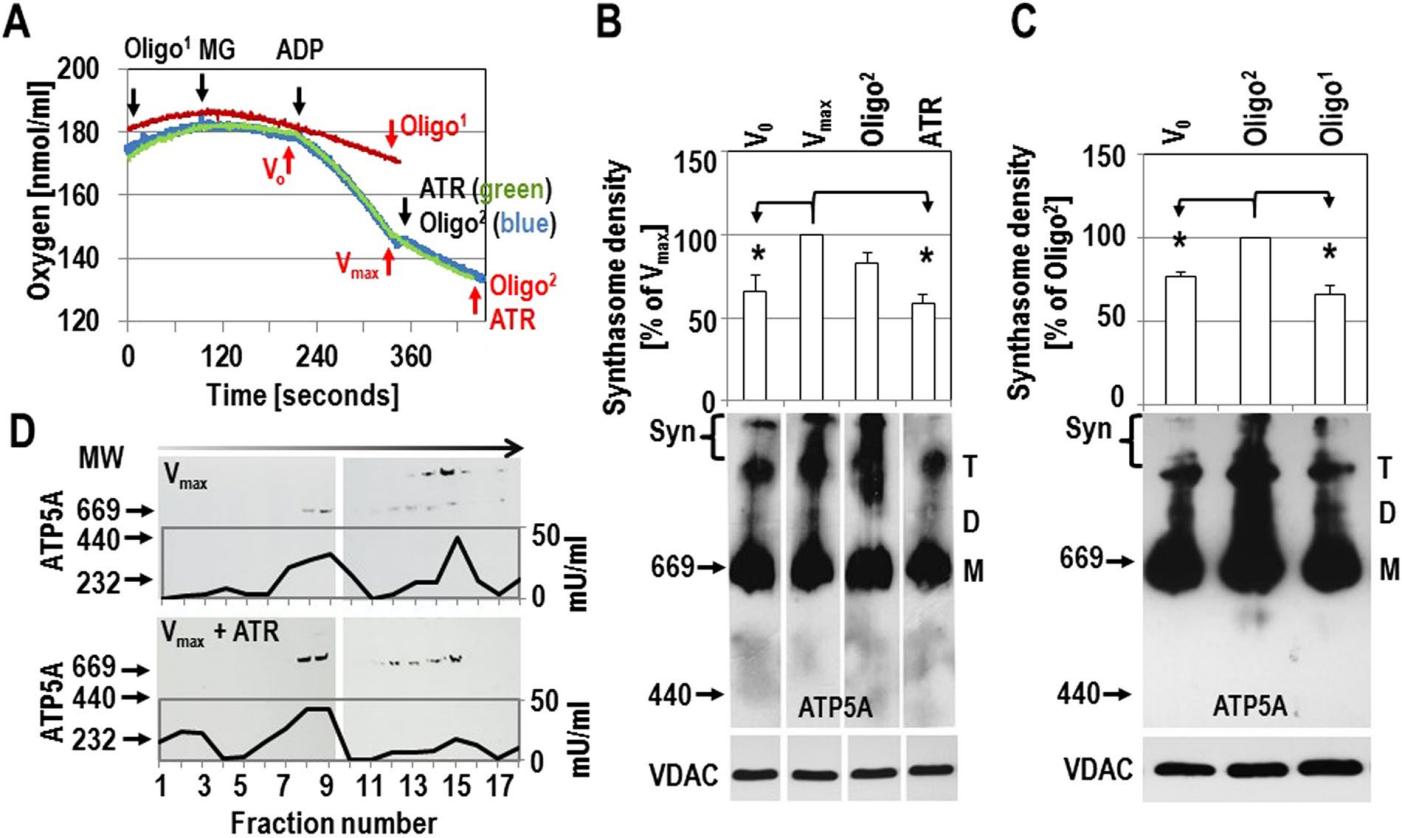
Respiration stimulates the formation of synthasomes. (**A**) Oxygraph recordings; black arrows indicate the addition of substrates or drugs (MG: (3 mM malate/5 mM glutamate), 1 mM ADP, 0.1 mM atractyloside (ATR), or 2 µg/ml oligomycin (Oligo1 when added before V_max_ or Oligo^2^ when added at V_max_) into the chamber, generally every 2 minutes. Red arrows indicate when samples were taken for CN electrophoresis. (**B**) Synthasome levels are highest during V_max_ (set as 100%) compared to V_0_ and ATR (n = 6), while oligomycin (Oligo^2^) had no significant effect (n = 3). (**C**) Addition of oligomycin at V_max_ (Oligo^2^) did not impact synthasome assembly but the addition of oligomycin before stimulation of OXPHOS (Oligo^1^) prevented the assembly of synthasomes. For B, C: Top panels show densitometric quantification (*p ≤ 0.05 compared to V_max_ by ANOVA), and middle panels represent native IB where the area indicated by Syn (synthasome) was scanned for densitometric analysis. The bottom panels show denaturing immunoblots for VDAC to demonstrate equal loading of samples. M, D and T refer to monomers, dimers and tetramers of ATP synthase. (**D**) Mitochondria at V_max_ (top panel) and after inhibition of V_max_ with 0.1 mM ATR (lower panel) were solubilized with 2 µg lauryl maltoside per µg protein and synthasomes and separated by sucrose-gradient centrifugation. Each fraction was examined by CN IB for ATP5A and measured for ATP synthase activity (n = 2, black lines on blots). MW in the gradient increases from left to right (direction of arrow) and fractions 9 and 13 had the highest absorbance readings for the MW marker proteins thyroglobulin (669 kD) and blue dextran 2000 (2,000 kD), respectively. Positions of MW markers (in kDa) for CN IBs are presented to the left of IBs.

The plasticity model suggests that ETC supercomplex assembly and disassembly is a dynamic process, but the mechanisms that control this are not understood. Therefore we tested whether the generation of synthasomes is controlled by the bioenergetic need of the mitochondria to produce energy. Mitochondria were isolated from adult mouse hearts and the respiratory control ratio (defined as the ratio of V_max_/V_0_^17^) was between 6 and 10 in all experiments, demonstrating normal oxidative function. Samples were taken for CN electrophoresis at V_0_ (substrate mediated respiration using malate and glutamate), after the addition of ADP to achieve maximal respiration (V_max_), and after the addition of inhibitors of ETC activity (Fig. 2A).

Monomers of ATP synthase were always detected in mitochondria from WT hearts. In addition, dimers, tetramers and higher molecular weight protein complexes, the synthasomes (Syn) were detected by IB for ATP5A (Figs. 2B, S3A). The presence of these synthasomes was measured by densitometry of IBs and was significantly higher at V_max_, with a higher ratio of synthasomes to monomers, compared to resting conditions (V_0_, Figs. 2B, S3A, S5A). Synthasomes were not significantly affected by the ATP synthase inhibitor oligomycin when it was added after V_max_ was obtained (Fig. 2A–C (Oligo^2^), S3A, S5A). However, inhibiting ATP synthase with oligomycin before stimulating OXPHOS prevented the formation of synthasomes at V_max_ and kept them at V_0_-levels (Figs. 2A,C, S3B (Oligo^1^)).

To further demonstrate the dynamic nature of synthasome assembly, we added 0.1 mM atractyloside (ATR), which inhibits ANT and shifts mitochondrial respiration back to V_0_. This led to a breakdown of the high MW synthasomes and an increase of monomers (Figs. 2B, S3A (n = 5, ATR), S5A). The presence of monomers and dimers of the ATP synthase indicates that the observed differences were not due to a failure to isolate and extract the synthasome (n ≥ 10, Figs. 2B,C, S3A, S3B), but to dynamic transitions between oligomeric states of assembly of the ATP synthase (Fig. S5 B). Furthermore, we did not see significant changes in levels of dimers or tetramers or the ratio of these to synthasomes or monomers (Figs. 2B, S3A, and S5B).

To complement these experiments, we separated synthasomes by sucrose density centrifugation (Fig. 2D). Each fraction was analyzed for the presence of ATP synthase by the oligomycin-sensitive enzymatic activity and by CN electrophoresis. In mitochondria at V_max_, ATP synthase activity was detectable in two peaks around fractions 9 and 15. CN electrophoresis showed that fractions 8 and 9 contain only the ATP synthase monomers, while fractions 14 and 15 contained synthasomes. However, treatment with ATR (Fig. 2D, V_max_ + ATR lower panel) decreased ATP synthase activity in fractions 14–16, and virtually eliminated the detection of synthasomes in all fractions. Meanwhile, there was no effect on the detection of ATP synthase around fraction 9. ATR treatment also caused an additional activity peak at a relatively low molecular weight (fractions 1–3), which was not sensitive to oligomycin and where ATP synthase was not detected by IB.

These data indicate that assembly and disassembly of synthasomes is a dynamic process, which is regulated by the activity of the ETC and ATP synthase.

### Synthasomes disassemble upon opening of the permeability transition pore

**Figure 3:**
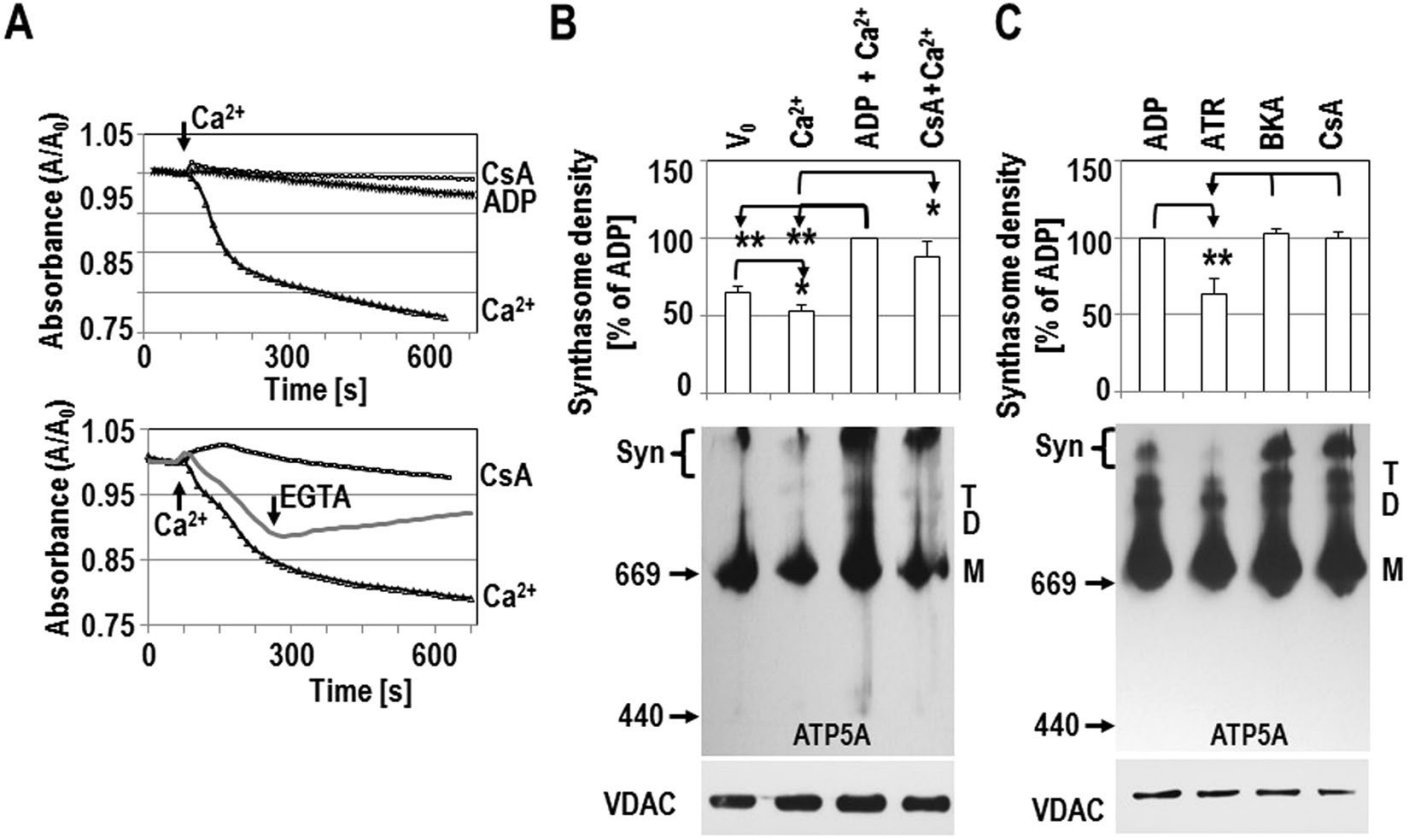
Permeability transition leads to disassembly of synthasomes. A: Mitochondrial PT was induced by 60 µM free Ca^2+^ (arrow) and was inhibited by 0.5 mM ADP or 200 nM CsA (top panel) or reversed after the addition of 250 µM EGTA (arrow; bottom panel). Onset of PT is presented as absorbance (**A**) over absorbance at time of Ca^2+^ addition (A_0_). (**B**) Densitometric analysis shows Ca^2+^-induced PT decreased synthasome levels and that this is inhibited by 0.5 mM ADP and 200 nM CsA (n = 7); changes are calculated relative to the signal in the presence of ADP (*p ≤ 0.05, **p ≤ 0.03 by ANOVA). Middle panel shows a representative CN blot. (**C**) 0.5 mM ADP, 10 µM bongkrekic acid (BKA) and 200 nM CsA inhibit PT and preserve the synthasome, while ATR mediates PT and decreases synthasome levels (n = 4). PT inhibitors or inducers were directly added to mitochondria in isotonic EGTA-free mannitol sucrose buffer in the absence of substrate or Ca^2+^. In the middle panels of B and C, monomers (M), dimers (D), tetramers (T), and synthasomes (Syn) of the ATP synthase were labeled using anti-ATP5A. Position of MW markers are indicated on the left. Denaturing immunoblots for VDAC below each blot demonstrated equal loading of samples.

Opening of the PTP uncouples ETC activity and oxygen consumption from ATP production, and recent data suggest that the PTP lies within ATP synthase^19,21,25^. To determine if opening of the PTP affects synthasome assembly, we treated isolated adult heart mitochondria with 60 µM free Ca^2+^ (240–300 nmol/mg protein), a concentration that opened the PTP and caused osmotic swelling (Fig. 3A, top panel). This PTP opening can be blocked by the PTP inhibitors CsA and ADP and can be reversed by the addition of the Ca^2+^ chelator EGTA (Fig. 3A). The dynamics of synthasome assembly were then evaluated using CN electrophoresis (Figs. 3B, S4A, S5B), as above.

Mitochondrial permeability transition (PT) is generally initiated in the presence of substrate (malate/glutamate) and phosphate but in the absence of magnesium (Mg^2+^), which are conditions similar to V_0_ and low levels of synthasomes (Figs. 3B, S4A, S5C, V_0_). The addition of Ca^2+^ to induce PT caused a small, but significant decrease of ATP synthase containing synthasomes, although changes in the ratio of synthasomes to monomers did not reach significance (Figs. 3B, S4A, S5C; V_0_ versus Ca^2+^). However, pre-incubating heart mitochondria with the PTP inhibitor ADP prevented swelling (Fig. 3A) and yielded the highest levels of synthasomes, the highest ratio of synthasomes to monomers, and a significant increase in the tetramer to monomer ratio(Figs. 3B, S4A, S5C, ADP). This may also occur due to the ability of ADP to initiate V_max_ (Fig. 2B). Therefore, when we also incubated mitochondria with 200 nM CsA in to inhibit the PTP, synthasome detection was equally high (Figs. 3B, S4A, S5C).

To further compare the effects of inhibitors or activators of the PTP on synthasome assembly, mitochondria were incubated with ADP, ATR, bongkrekic acid (BKA) and CsA in the absence of any substrate, phosphate or Ca^2+^ (Figs. 3C, S3B). In heart mitochondria from WT mice, the PTP inhibitors CsA and BKA were equally effective as ADP in increasing synthasome assembly by themselves, while the PTP inducer ATR kept the level of synthasomes low (Figs. 3C, S4B). As ATR and BKA both inhibit ANT, these effects are likely not due to the inhibition of ATP/ADP transport but are instead due to their opposing effects on the PTP and/or synthasome assembly. Overall, these data show that favoring PTP opening results in disassembly of synthasomes, while inhibiting the PTP enhances synthasome assembly.

### Cyclophilin D regulates synthasome assembly

The data presented above suggest that synthasome assembly and PTP opening are inversely proportional. CypD is the most accepted regulator of the PTP. However, its interaction with the ATP synthase (Fig. 1C and D) suggests that CypD might also regulate the assembly and stability of synthasomes. Therefore, we sought to determine how the synthasomes respond to OXPHOS and conditions that resemble PT using heart mitochondria from adult CypD KO mice.

**Figure 4:**
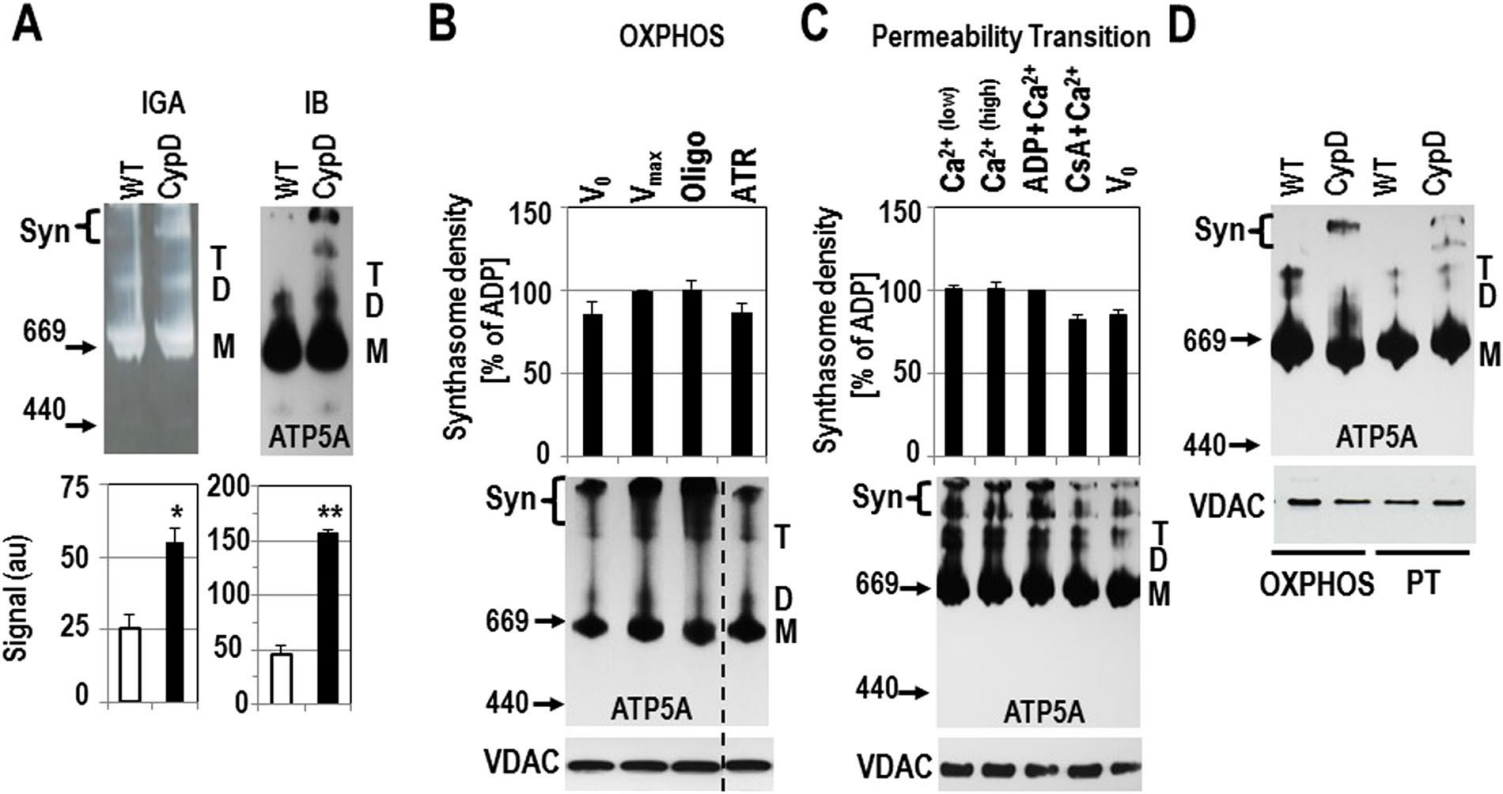
Synthasome levels are higher but less dynamic in hearts from CypD KO mice. (**A**) ATP synthase in-gel assay (IGA) and immunoblotting (IB, for ATP5A) after CN PAGE demonstrated more synthasomes (Syn) in CypD KO hearts compared to WT hearts (upper panel) and was confirmed by densitometric quantification (lower panel, arbitrary units (au)) (n = 4, *p ≤ 0.05, **p ≤ 0.001 by T-test). Note that the ATP synthase IGA results a white reaction product, so the shading is correct. (**B**) and (**C**) No significant changes were observed in synthasome levels in CypD KO mitochondria during OXPHOS (B, n = 3) and Ca^2+^-induced PT (C, n = 4). The experimental conditions are indicated. (Abbreviations and concentrations: V_0_: 3 mM malate/5 mM glutamate, V_max_: 1 mM ADP, ATR: 0.1 mM atractyloside, Oligo: 2 µg/ml oligomycin (added at V_max_), CsA: 200 nM cyclosporin A, Ca^2+ (low)^: 60 µM, Ca^2+ (high)^: 1 mM). No groups were significantly different by ANOVA. Dashed vertical line in IBs in B indicate moving the ATR lane from the same IB for presentation. (**D**) WT and CypD KO mitochondria run on the same gel featuring the experimental conditions in B (OXPHOS; + Mg^2+^) and C (PT; no Mg^2+^) and show patterns similar to B and C, respectively. Synthasome containing areas (Syn) in each lane were scanned for analysis. M, D, and T refer to monomers, dimers and tetramers of the ATP synthase, respectively. Positions of MW markers (in kDa) for CN IBs are presented to the left of IBs. In B-D denaturing immunoblots for VDAC below each blot demonstrated equal loading of samples.

We found significantly more high-molecular weight synthasomes by IB and IGA in resting heart mitochondria from CypD KO mice compared to WT mice (Figs. 4A, S6A, S6B). No CypD was detectable in the synthasomes of heart mitochondria from CypD KO mice by SDS electrophoresis (Fig. S6 C). Interestingly, when OXPHOS was stimulated in heart mitochondria from CypD KO mice, synthasome assembly was not as dynamic as observed in WT mitochondria: the level of synthasomes was similarly high at rest (Figs. 4A, S6A, S6B), V_0_ and V_max_ (Figs. 4B, S7A, S8A). In contrast to WT mice, ATR had no significant effect on synthasome disassembly (Figs. 4B, S7A, S6B). These data support the idea that the absence of CypD in these mitochondria prevents an immediate, dynamic response to bioenergetic requirements.

Furthermore, inducing PT with Ca^2+^ had no effect on synthasome levels in the absence of CypD (Figs. 4C, S5B). Both 60 µM Ca^2+^, which opens the PTP in WT samples but not in CypD KO samples^19,29^, and 1 mM Ca^2+^ (4–5 µmol/mg protein) which is required to cause PT in CyPD KO samples^19,28,29,31^ had no effect on synthasome levels in CypD KO mitochondria (Figs. 4C, S7B, S8B) or the level of monomers (Fig. S8 A). In addition, the typical PTP inhibitors ADP and CsA had no significant effect on the state of the synthasome or synthasome to monomer ratio (Figs. 4C, S7B, S8C). We also saw no changes in the levels of tetramers in these experiments, but we were not able to evaluate dimer levels, as they were inconsistently observed at only low levels. We noticed a different banding pattern of ATP5A immunolabeling in OXPHOS and PT experiments using CypD KO hearts. In OXPHOS experiments monomers and oligomers/synthasomes dominated, while in PT experiments dimers and tetramers of ATP synthase were more prominent. These differences may be due to the experimental conditions such as the presence of Mg^2+^ in the assay medium (Figs. 4D, S7C) or the time to complete an OXPHOS experiment (6–8 minutes) versus a PT experiment (15–20 minutes).

**Figure 5:**
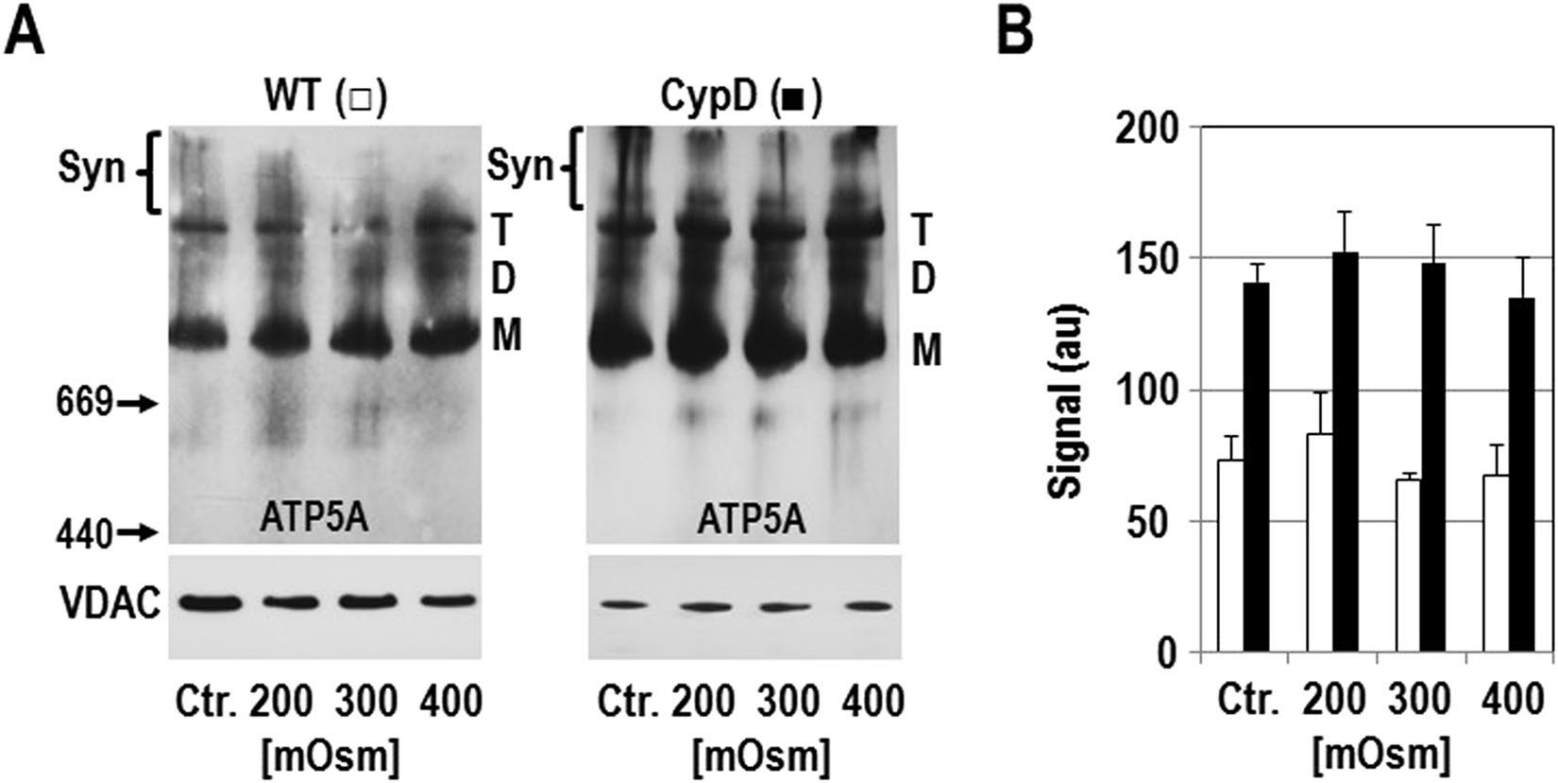
Formation of synthasomes during V_max_ is not due to changes of the mitochondrial matrix osmolarity. (**A**) Isolated mitochondria from WT and CypD KO hearts (250 µg) were exposed for 15 minutes to a buffer (0.5 ml) where the concentration of mannitol and sucrose were adjusted so that the final osmolarity was normal (300 mOsm), low (200 mOsm), and high (400 mOsm)^34^ or in EGTA-free mannitol/sucrose buffer (Ctr). M, D, T, and Syn refer to monomers, dimers, tetramers, and synthasomes of ATP synthase, respectively. Positions of MW markers (in kDa) for CN IBs are presented to the left of IBs. Denaturing immunoblots for VDAC below each blot demonstrated equal loading of samples. (**B**) Quantification shows no effect of the osmotic conditions on the prevalence of synthasomes in WT (□, n = 4) and CypD KO (■, n = 3) mitochondria as assessed by ANOVA.

To exclude the possibility that the dynamic assembly of synthasomes is due to a transition of mitochondria from an orthodox (relaxed matrix compartment) to a condensed state (contracted matrix compartment) after the addition of ADP^32,33^, we subjected mitochondria from WT and CypD KO hearts to an osmotic challenge by varying the concentration of mannitol and sucrose in the buffer^34^. However, a change of osmolarity alone did not cause any changes in the presence of synthasomes in WT or CypD KO mice (Figs. 5, S9). We conclude that the dynamic changes in heart synthasomes are not due to changes in the matrix conformation or osmotic stress, but arise as a consequence of the bioenergetic conditions caused by OXPHOS, initiation of PT and the ability of CypD to regulate ATP synthase containing synthasomes supercomplexes.

### Synthasome assembly correlates to CypD activity in different tissues

**Figure 6:**
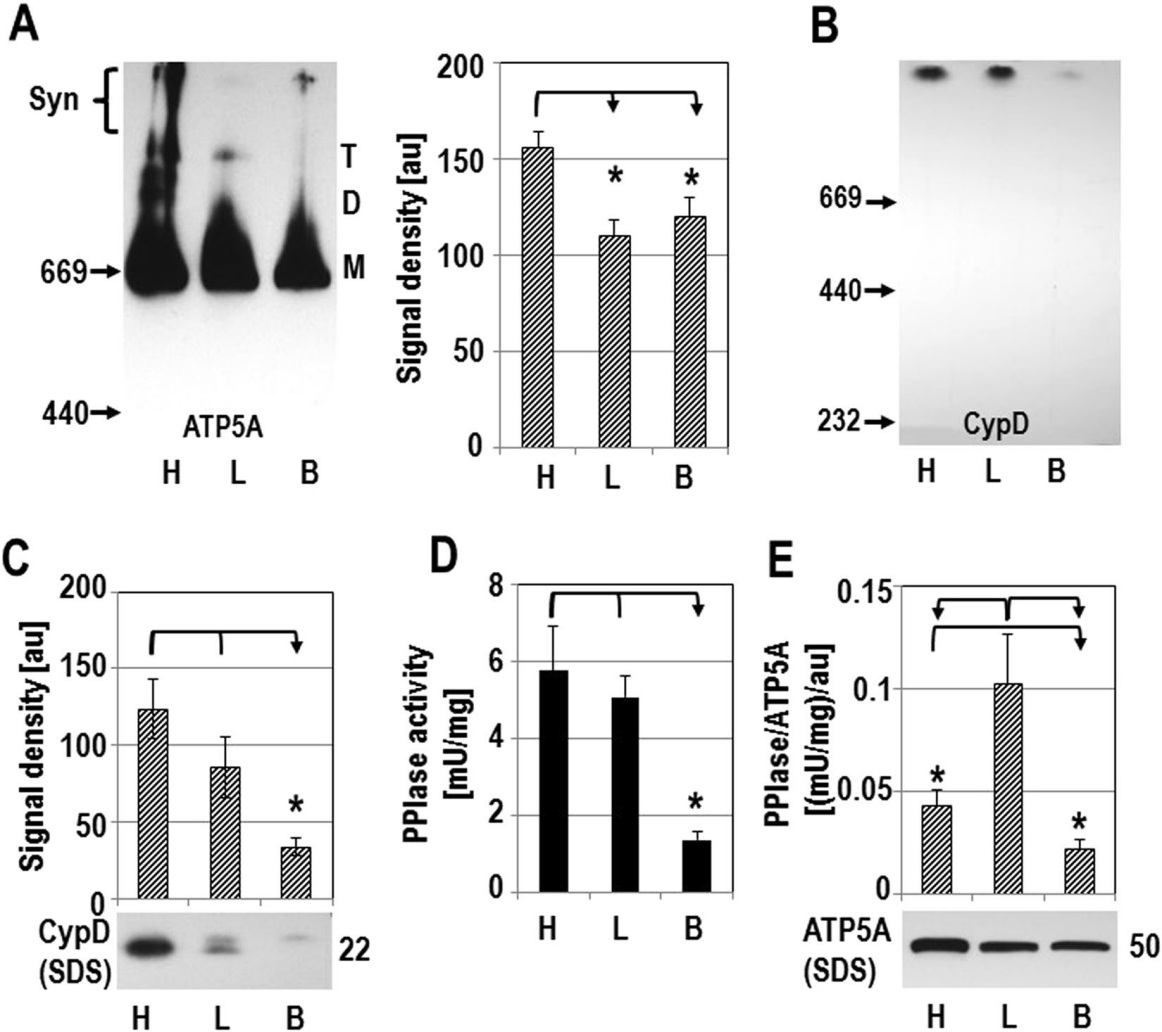
CypD activity correlates with synthasome levels in heart, liver and brain mitochondria. (**A**) CN PAGE of heart (H), liver (L) and brain (B) mitochondria show distinct patterns of ATP synthase assembly (left, M, D, T, and Syn refer to monomers, dimers, tetramers, and synthasomes respectively). Quantification demonstrates differences in synthasome levels between tissues (right, n = 7, p ≤ 0.01). (**B**) Less CypD is found in synthasomes of brain compared to heart and liver mitochondria (n = 5; p ≤ 0.03). (**C**) Total expression of CypD is lower in the brain compared to liver and heart in denaturing IBs (n = 7, p ≤ 0.01). (**D**) Cyclosporin A-sensitive peptidyl-prolyl *cis*/*trans* isomerase (PPIase) activity is lower in brain compared to heart and liver mitochondria (n = 4, p ≤ 0.005). (**E**) When normalized to total expression of ATP5A, the specific activity of PPIase is higher in liver compared to heart and brain mitochondria (n = 5; p ≤ 0.02). All comparisons by ANOVA.

Mitochondria from heart, liver and brain have different bioenergetic requirements, which could result in different levels of synthasomes in each tissue. As described throughout this work, ATP synthase in the heart is assembled into monomers, dimers, tetramers, and high molecular weight synthasomes. Densitometry indicated fewer synthasomes in native immunoblots from liver and brain mitochondria (Figs. 6A, S10A). We found that liver mitochondria contained mostly monomers and tetramers, while signals for dimers and synthasomes were faint (Figs. 6A, S10A, see also^35^). Brain mitochondria however contained mostly monomers and synthasomes, but no dimers and few tetramers (Figs. 6A, S10A, see also^35^). CypD was found only in synthasomes of mitochondria from these tissues, but significantly less CypD was found in synthasomes from brain mitochondria (Figs. 6B, S10B). The expression of CypD (Figs. 6C, S10B) and its PPIase activity (Fig. 6D) were also markedly lower in brain mitochondria compared to liver or heart. The specific activity of CypD (activity (Fig. 6D) divided by expression (Figs. 6C, S10B)) did not correlate with supercomplex levels in each tissue. However, the ratio of CypD activity relative to the expression of ATP5A in these tissues was significantly higher in liver mitochondria compared to heart or brain mitochondria, suggesting that a low ratio of active CypD to total ATP synthase favors synthasome assembly in heart and brain mitochondria (Figs. 6E, S10C). Of note, using SDS-PAGE, we consistently found two bands labeled for CypD in heart and liver samples and only the higher molecular weight band of CypD in brain (Fig. 6C). The significance of this is unclear, but could be due to post-translational modifications^36^. Together these results support the premise that the requirements for the ATP synthase to assemble into synthasomes are tissue-specific and dependent on the activity of CypD.

## Discussion

**Figure 7:**
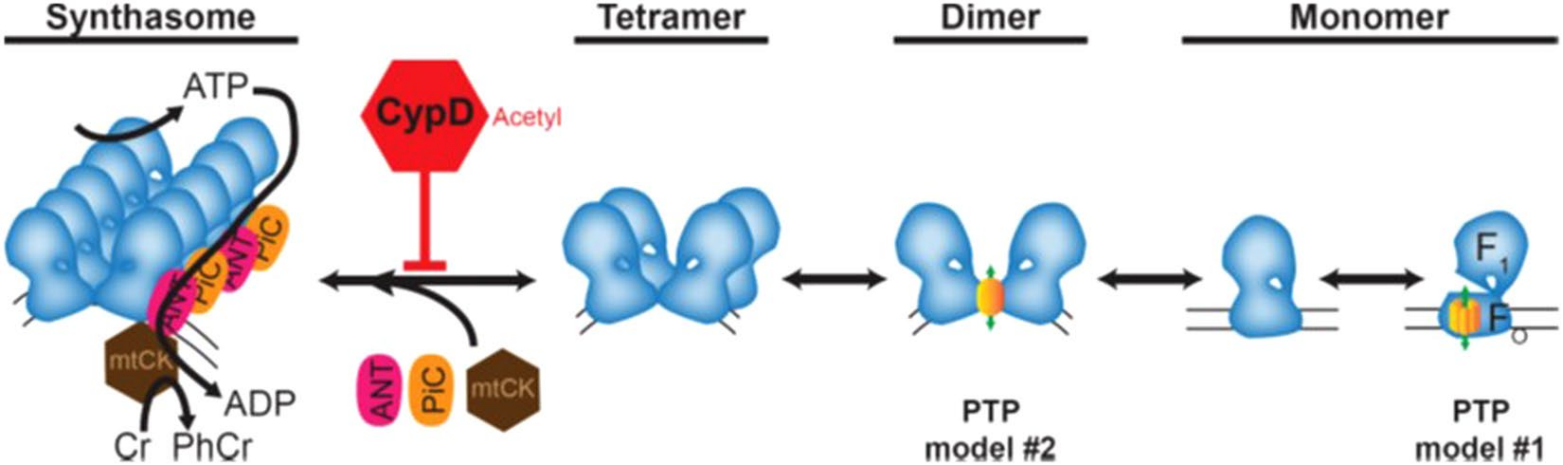
A model of CypD’s regulation of synthasome assembly and PTP formation. Monomers of the ATP synthase (blue complex) assemble into dimers and tetramers, which then can assemble with the ANT, PiC, and mtCK into high order oligomers (synthasomes) depending on the bioenergetic needs of the cardiomyocyte. CypD, perhaps activated by acetylation, inhibits synthasome assembly and increases the number of monomers, which may disassemble to expose the c-subunit ring (PTP model #1), and dimers (PTP model #2) (see Discussion for further details).

The data reported here add to the growing body of evidence that ETC supercomplexes are present in mitochondria of mammals, cell lines, and plants to generate micro-compartments for efficient electron transfer and ATP production and exchange^11,16,37^. Furthermore, we demonstrate that the formation of synthasomes in hearts from WT mice (C57BL/6N) is an immediate and dynamic process, which is favored by oxidative activity and by conditions that prevent PT (Figs. 2 and 3 and corresponding Supplement Figures). The current literature discusses three models of the functional ETC: a solid state model, a fluid model, and a hybrid, the plasticity model^8,38^. Our results show that, in heart mitochondria, ATP synthase assembles and disassembles into synthasomes depending on the bioenergetic needs (OXPHOS, PT), supporting the plasticity model for this protein complex (Fig. 7). Furthermore, our results indicate for the first time that CypD is a key regulator in maintaining this plasticity as it is not observed in heart mitochondria from CypD KO mice.

Mitochondria are very dynamic organelles. The mitochondrial network undergoes constant changes due to fission and fusion^39,40^. In addition, the mitochondrial ultrastructure depends on the respiratory state: respiring mitochondria are condensed with tightly packed elongated cristae membranes, while resting mitochondria are in a relaxed configuration^34^. It has been previously shown that ribbons of oligomeric ATP synthase (e.g., synthasomes) mold the cristae into elongated, tubular structures and that this increases the efficiency of the utilization of the proton gradient and the transmission of energy equivalents into the cytoplasm^13,14^. Thus, the dynamics displayed by the mitochondrial network appear to be directly linked to the morphology of the cristae membrane and, as we show here, the formation of ATP synthase containing supercomplexes.

In the adult heart, monomers of the ATP synthase assemble into dimers, tetramers and oligomers^16,41^. We showed that the functional synthasomes also contain ANT, PiC and mtCK, therefore connecting ATP producing and ATP consuming pathways into one bioenergetic active unit (Figs. 1, 2 and 7). The presence of synthasomes is highest in actively respiring mitochondria, a bioenergetic condition where the TCA cycle is stimulated and ADP is used by the ATP synthase to generate ATP (Fig. 2), supporting the idea that these supercomplexes of ATP synthase maximize OXPHOS efficiency. This also suggests a positive feedback loop involving respiration, synthasome formation, and mitochondrial structure: as respiration increases, synthasomes form to increase bioenergetic efficiency by both folding the inner membrane into a more efficient structure and bringing together a massive macromolecular complex that increases energy transmission to the cytoplasm.

Opening of the PTP is a devastating event for the cell if it is irreversible. However, reversible PT is thought to be a stress response that protects mitochondria and the cell^42^. Previous work suggests that a bioenergetic activity is related to decreased susceptibility to PT^27^, but how this occurs is not currently understood. We show that, in the heart, inducing PT decreases synthasome levels, while inhibiting PT increases them. This explanation provides a mechanism that links synthasomes and PT: synthasome assembly would favor ATP production in the matrix, which is transported by ANT into the intermembrane space to produce creatine phosphate by mtCK. This would lead to high concentrations of the PT inhibitor ADP in a micro-compartment within the synthasome, therefore preventing PTP opening.

However, the activity of CypD reveals another mechanism that links synthasome assembly and PT. We and others have shown that the PTP is within the ATP synthase^19–22,24,25^, thereby connecting the PTP to the synthasome. CypD, a well-established regulator of the PTP, appears to be a key regulator of the transition of ATP synthase into the PTP. Except for mtCK, which is not present in liver mitochondria, CypD binds to all proteins of the synthasome: OSCP of the catalytic domain of ATP synthase^43^, the ANT^44,45^ and the PiC^46^. Our data demonstrate that inhibition or deletion of CypD increases the stability of synthasomes in heart mitochondria and prevents the dynamic regulation of these structures (Fig. 4).

Furthermore, we found that differences in CypD activity may regulate synthasome assembly in different tissues. The presence of synthasomes varied markedly between the heart, liver and brain. The heart contained ATP synthase at all levels of assembly, while the liver contained predominately monomers and tetramers and the brain contained monomers and synthasomes. Overall, the pattern of high molecular weight ATP synthase molecules in brain mitochondria from WT mice resembled that of heart mitochondria from CypD KO mice (compare Figs. 4B and 6A), and CypD levels were lowest in brain compared to heart and liver. In contrast, liver mitochondria have only a few synthasomes and contain high levels of CypD. Although the relative amounts of CypD in heart and liver are the same, our data suggest that the amount of active CypD relative to the total amount of ATP synthase present in each organ controls assembly of synthasomes. We did not investigate the dynamic regulation of CypD activity in these tissues, but CypD is regulated by a variety of post-translational modifications, including acetylation and phosphorylation, that suggest a sophisticated regulation. In particular, acetylation is known to activate CypD^36,47,48^ while increased respiratory activity promotes deacetylation that may render CypD inactive: increased respiration decreases levels of acetyl-CoA, the source of the acetyl group for protein acetylation, while also increasing levels of NAD^+^, thus increasing the activity of the mitochondrial deacetylase, SIRT3^49^.

Overall, these data suggest a model, in which active CypD may bind to and limit the assembly of synthasomes (Fig. 7). This model is consistent with the previously observed findings that increased OXPHOS activity and the presence of elongated cristae are associated with decreased PTP opening and cell death^12^. Intriguingly, this model predicts that CypD’s control of PTP opening could be secondary to its effects on synthasome assembly. How the ATP synthase creates the PTP is currently a matter of great debate, but our model is consistent with the current competing theories of the origin of the PTP within the ATP synthase. We and others have proposed that the central pore of the membranous c-subunit ring of the ATP synthase can be exposed to become the pore of the PTP (PTP model #1 in Fig. 7)^19–22,24,27^. In contrast, others have proposed that the membranous region around ATP synthase dimers creates the PTP (PTP model #2 in Fig. 7)^25^. Commentaries on these data^23,50–52^ suggest that the story is more complicated and implicate the other components (ANT, PiC, mtCK) of the synthasome as regulatory players, so the results we report here are not inconsistent with either of these theories, although we note that three recent reports dispute the claim that ATP synthase creates the PTP^53–55^. However, the lack of dynamic changes in dimer levels supports model #1 and not model #2, but further work will be needed to resolve this issue.

In summary, these data suggest a model where CypD plays an important role in the regulation of mitochondrial energy production and transmission to the cell (Fig. 7). De-activation of CypD would promote the assembly of synthasomes for efficient OXPHOS and energy transfer to the cytoplasm, while also decreasing the likelihood of synthasome disassembly and the probability of PTP opening. In contrast, activation of CypD would limit synthasome assembly, decreasing the efficiency of respiration and altering inner membrane structure, while increasing the probability of PTP opening. This dynamic is lost in CypD KO mice, where synthasomes are permanent structures that are independent of the bioenergetic state. This may explain why cardiac mitochondria from CypD null mice are bioenergetically more active and less responsive to increased energetic demand, a phenomenon that has been attributed in part to increased levels of matrix Ca^2+^ ^31^. The detailed mechanisms, by which this occurs and whether respirasome assembly is controlled by similar mechanisms, remain to be determined.

## Methods

### Animals

All experiments were performed using C57BL/6N mice (wild type) and CypD knockout mice in a C57BL/6N background^28^. Mice were anesthetized with CO_2_ prior to cervical dislocation and all procedures were in strict accordance with the Division of Laboratory Animal Medicine, at the University of Rochester, and in compliance with state law, federal statute, and NIH policy. The protocol was approved by the Institutional Animal Care and Use Committee of the University of Rochester (University Committee on Animal Resources (UCAR)).

### Isolation of mitochondria

Mitochondria were isolated from adult mouse heart, liver and brain by differential centrifugation^56^. Mitochondria were washed and minced in ice-cold mannitol/sucrose-buffer (225 mM mannitol, 10 mM sucrose, 0.5 mM EGTA, 10 mM HEPES, pH 7.4) by differential centrifugation at 500 and 10,000 g. The final sediment was resuspended in EGTA-free mannitol/sucrose buffer. The isolated mitochondria are highly enriched in the inner mitochondrial membrane protein ANT and cytochrome c, but devoid of the cytosolic glyceraldehyde dehydrogenase (GAPDH) (Supplement Fig. S11).

### Immunoprecipitation of mitochondrial supercomplexes

200 µg of isolated mitochondria were diluted in phosphate buffered saline (PBS) to 0.5 mg/ml, protease inhibitor cocktail (P8340 from Sigma) was added, and the membranes were solubilized with 1 mM lauryl-maltoside in PBS for 20 minutes on ice. After centrifugation to remove tissue fragments, mitochondrial supercomplexes were incubated with antibodies (sources listed below) and precipitated with protein G agarose (Roche Bioscience). Proteins were eluted from the beads by boiling samples for 5 minutes in SDS sample buffer (100 mM Tris base pH 6.8; 14 mM SDS, 3 mM bromophenol blue, 12 mM β-mercaptoethanol).

### Stimulation of ETC activity

Respiratory activity of isolated mitochondria (200–300 µg protein suspended in respiration buffer: 120 mM KCl, 65 mM mannitol, 35 mM sucrose, 5 mM KH_2_PO_4_xK_2_HPO_4_ (pH 7.4), 20 mM Tris (pH 7.4) and 5 mM MgCl_2_) was measured with a Clark oxygen electrode (Hansatech, Amesbury MD) at room temperature^17^. V_max_ was inhibited either by the addition of 2 µg/ml oligomycin (Oligo) or 100 µM ATR. At the end of each experiment, samples were immediately transferred into ice-cold micro-tubes and centrifuged for 5 minutes at 13,000 g. The mitochondrial sediment was then prepared for CN electrophoresis.

### PT (mitochondrial swelling) assay

PT was measured as the decrease of absorbance at 540 nm at room temperature^19^. Mitochondria were suspended in PT buffer (120 mM KCl, 65 mM mannitol, 35 mM sucrose, 5 mM KH_2_PO_4_xK_2_HPO_4_ (pH 7.4), 20 mM Tris (pH 7.4), 5 mM glutamate and 3 mM malate). PT was initiated by the addition of Ca^2+^ and inhibited by pre-incubating isolated heart mitochondria for 5 minutes with 200 nM CsA or 0.5 mM ADP in PT buffer. 200–250 µg of mitochondrial protein were used per experiment. Free Ca^2+^ concentrations in the presence of EGTA were calculated using MaxChelator from the University of Stanford (http://www.stanford.edu/~cpatton/maxc.html) (60 µM and 1 mM Ca^2+^ are equivalent to 240–300 nmol/mg and 4–5 µmol/mg protein, respectively. At the end of each experiment, samples were prepared for CN electrophoresis, as above.

### Separation of ETC complexes by sucrose density centrifugation

All steps were performed on ice and were similar to^9,10^. Briefly, 1.75 M sucrose was dissolved in Tris-buffer (20 mM TrisHCl, pH 7.4, 20 mM KCl, protease inhibitor cocktail P8340). Tris-buffer was also used to dilute the 1.75 M sucrose solution to 1.5 M, 1 M, 0.6 M and 0.3 M. A gradient was assembled by layering 1.5 ml of each sucrose concentration into centrifugation tubes, beginning with 1.75 M sucrose at the bottom and finishing with 0.3 M followed by the mitochondria as the final layer. After centrifugation at 125,000 g for 18 hours in a swing bucket rotor the gradient was pipetted into 18 fractions of 500 µl. The absorption peaks of the molecular weight markers for thyroglobulin (669 kD) and Blue dextran (2,000 kD) (GE Healthcare, Piscataway NJ)) were in fraction 9 and 13, respectively (n = 3).

### Native electrophoresis

Protein complexes from 20 µg of mitochondria (per lane) were separated on a 3–8% Clear Native (CN) gel^35^. Samples, obtained directly after completing an experiment or after not more than one cycle of freezing and thawing, were solubilized on ice with 2 µg lauryl-maltoside/µg protein in extraction buffer (50 mM NaCl, 50 mM imidazole/HCl, 2 mM 6-aminohexanoic acid, 1 mM EDTA, pH 7.0). After separation (200 V for 2 hours on ice), the protein complexes were either wet-transferred onto nitrocellulose or polyvinylidene fluoride (PVDF) membranes (25 V, 16 hours) or used for in-gel assays. Membranes were stained with Ponceau S after transfer and photographed. Synthasomes were identified by the detection of ATP5A and the position of ATP5A containing synthasomes is indicated by a bracket. An aliquot of each sample prepared for CN electrophoresis was used to verify protein loading of these blots by the detection of the voltage dependent anion channel (VDAC), a protein of the outer mitochondrial membrane, and is shown below CN blots. Except for 6B, CN blots show only protein complexes that are greater than 440 kD and all respective full length CN blots are shown in the supplement.

### Denaturing SDS electrophoresis

3–10 µg of mitochondrial protein were separated on a 16% polyacrylamide gel, followed by wet-transfer (80 V for 60 minutes on ice) onto nitrocellulose membranes.

### Densitometry

The signal intensity of the synthasomes was analyzed with Image J by densitometry scans^17^, as described. For densitometry the maximal signal intensity of the synthasomes in each experiment (always seen in the presence of ADP) was set as 100%. For some experiments arbitrary units (au) are used.

### ATP-synthase activity assays

ATP synthase activity was visualized in CN gels using published protocols for IGA^35,57^. In addition, ATP synthase activity was measured at room temperature spectrophotometrically by its ability to hydrolyze ATP according to^58^.

### Peptidyl-prolyl *cis/trans* isomerase activity assay

The activity of CsA-sensitive peptidyl-prolyl *cis/trans* isomerase activity was measured according to^59^. Briefly, mitochondria were added to an assay mix containing 50 µM *n*-succinyl-alanine-alanine-proline-phenylalanine-*p*-nitroanilide in 50 mM Tris-HCl, pH 8.0. The reaction was started with α-chymotrypsin (0.1 mg/ml) and the change of absorbance was followed at 390 nm. After an immediate fast increase/burst of absorbance due the cleavage of the trans form of *n*-succinyl-alanine-alanine-proline-phenylalanine-*p*-nitroanilide, the cis form of the peptide is accessible *cis/trans* isomerases.

### Osmolarity challenge

Isolated mitochondria from WT and CypD KO hearts (250 µg) were incubated on ice for 15 minutes in 0.5 ml EGTA-free mannitol/sucrose buffer (Ctr) or in a buffer where the concentration of mannitol and sucrose were adjusted so that the final osmolarity was normal (300 mOsm), low (200 mOsm), and high (400 mOsm)^34^. Samples were then centrifuged and the mitochondrial sediment was prepared for CN PAGE.

### Protein determination and protein loading controls

Protein concentrations were determined using a BCA protein assay from Pierce. From each sample used on CN electrophoresis, an aliquot was run on a SDS gel and labeled for VDAC to verify equal protein loading.

### Statistical analysis

Statistical analysis was performed using Prism (GraphPad, V 6.07). Data were analyzed for significance using ANOVA with Tukey’s post-hoc testing or unpaired, two tailed T-tests where appropriate, and p ≤ 0.05 was considered to be significant. All graphs are represented as mean +/− SE, and the number of individual experiments (n) for each data set is stated in the Figure legends.

### Antibodies used

Anti-ANT (adenine nucleotide translocase; detects isoforms 1,2 and 3, sc-9300 from Santa Cruz); anti-ATP5A (α- subunit, ab14748, Abcam); anti-ATP5G (c-subunit, ab 180149, Abcam) anti-ATP synthase immunocapture antibody (ab109867 Abcam); anti-complex 1 immunocapture antibody (ab109798, Abcam); anti-complex 3 immunocapture antibody (ab109862, Abcam); anti-CypD (cyclophilin D, ab 110324, Abcam); anti-GAPDH (MAB374 Chemicon) anti-mtCK (mitochondrial creatine kinase, sc-15169, Santa Cruz); anti-NDUFAB1 (ab96230, Abcam); anti-OSCP (oligomycin sensitivity conferring protein, sc-365162, Santa Cruz); and anti-VDAC (voltage dependent anion channel)/anti-porin 31HL (529534, Calbiochem).

### Data Availability

All data generated or analyzed during this study are included in this published article (and its Supplementary Information files).

## Electronic supplementary material

Supplementary Information
