# Supplementary material for: Cyclophilin D regulates the dynamic assembly of mitochondrial ATP synthase into synthasomes
Source: Sci Rep. 2017 Nov 3;7:14488. doi: 10.1038/s41598-017-14795-x (PMC5670235; doi:10.1038/s41598-017-14795-x)
Supplement: Supplementary file 1 — Supplementary Information [file 41598_2017_14795_MOESM1_ESM.pdf]

Cyclophilin D regulates the dynamic assembly of mitochondrial ATP synthase into synthasomes

Gisela Beutner [1], Ryan E. Alanzalon[1], George A Porter, Jr. [1,2,3]*

[1]Department of Pediatrics (Cardiology), [2]Department of Pharmacology and Physiology, [3]Department of Medicine (Aab Cardiovascular Research Institute), University of Rochester, Rochester, New York 14642, United States

[*]Correspondence: george_porter@urmc.rochester.edu

Corresponding author:
George A. Porter, Jr., MD, PhD
Associate Professor
University of Rochester Medical Center
Department of Pediatrics
Division of Cardiology
601 Elmwood Ave. Box 631
Rochester, NY 14642
USA
Email: george_porter@urmc.rochester.edu
Phone: (585) 276-4769

Fax: (585) 275-7436

Keywords: heart mitochondria, electron transport chain, ATP synthase, synthasomes, cyclophilin D

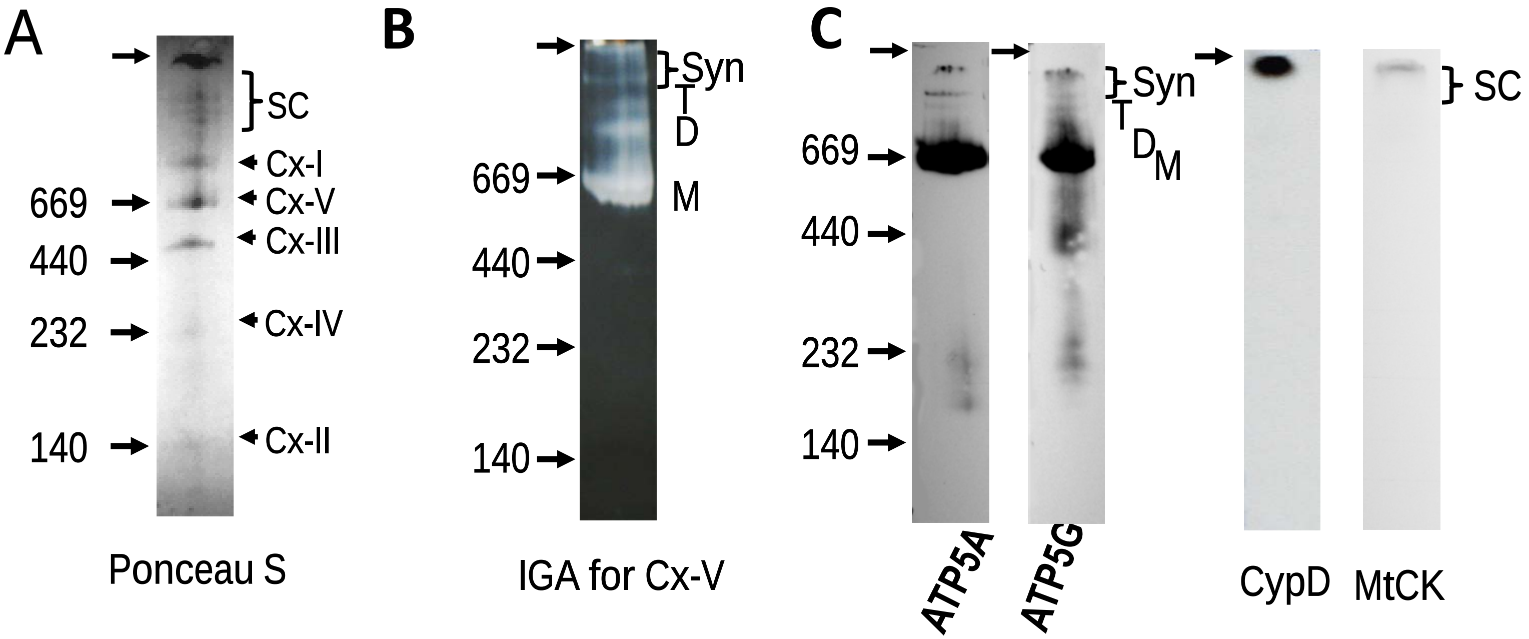

Supplement Figure 1-Beutner:

Supplement Figure 1: Full lanes of blots shown in Figure 1A, B, and C. A: Ponceau S staining of a CN PAGE after transfer onto nitrocellulose membranes shows a distinct pattern of monomeric ETC complexes (Cx) I, V, and III and supercomplexes (SC). B: Representative in-gel-assay (IGA; n = 8). C: Immunoblotting for ATP5A (n ≥ 10) and ATP5G (n = 1) demonstrate monomers (M), dimers (D), tetramers (T) and synthasomes (Syn) in CN gels. In parallel labeling, CypD (n=5) and mtCK (n=5) are present only in high molecular weight protein complexes. Arrows at the top of CN blots indicate the bottom of the well. The positions of the molecular weight (MW) markers (in kDa) are indicated by arrows on the left.

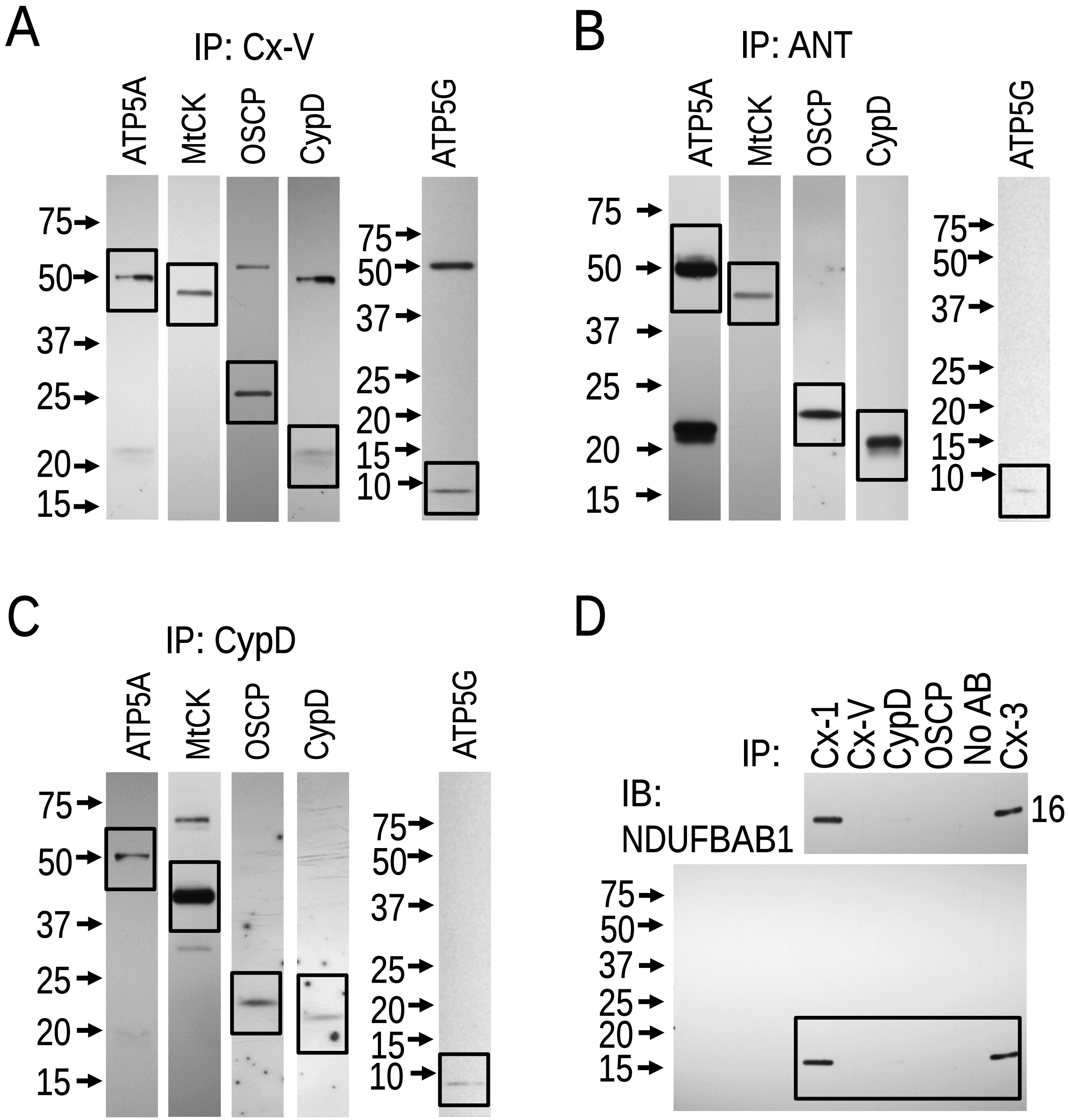

Supplement Figure 2-Beutner:

Supplement Figure 2: Full lanes of the IP bands shown in Figure 1D and 1E. A-C: Immunoprecipitation of the synthasome with antibodies against ATP synthase (Cx-V, n≥5, (A)), ANT (n=3, (B)) and CypD (n=2, (C)) followed by IB against ATP5A (n=5), mtCK (n=3), OSCP (n=2), CypD (n=3) and ATP5G (n=1). Please note that antibodies against ATP5A and CypD were used together. D: Precipitates obtained with antibodies against ATP synthase (Cx-V), OSCP, and CypD do not contain the subunit NDUFAB1 of Cx-I, while IP of Cx-I and Cx-III do (n=3). D: Full blot of Figure 1E. Precipitates obtained with antibodies against ATP synthase (Cx-V), CypD and OSCP do not contain the subunit NDUFAB1 of Cx-I, while IP of Cx-I and Cx-III do (n=3). Boxed areas indicate bands of interest. In all blots the positions of the molecular weight (MW) markers (in kDa) are indicated on the left.

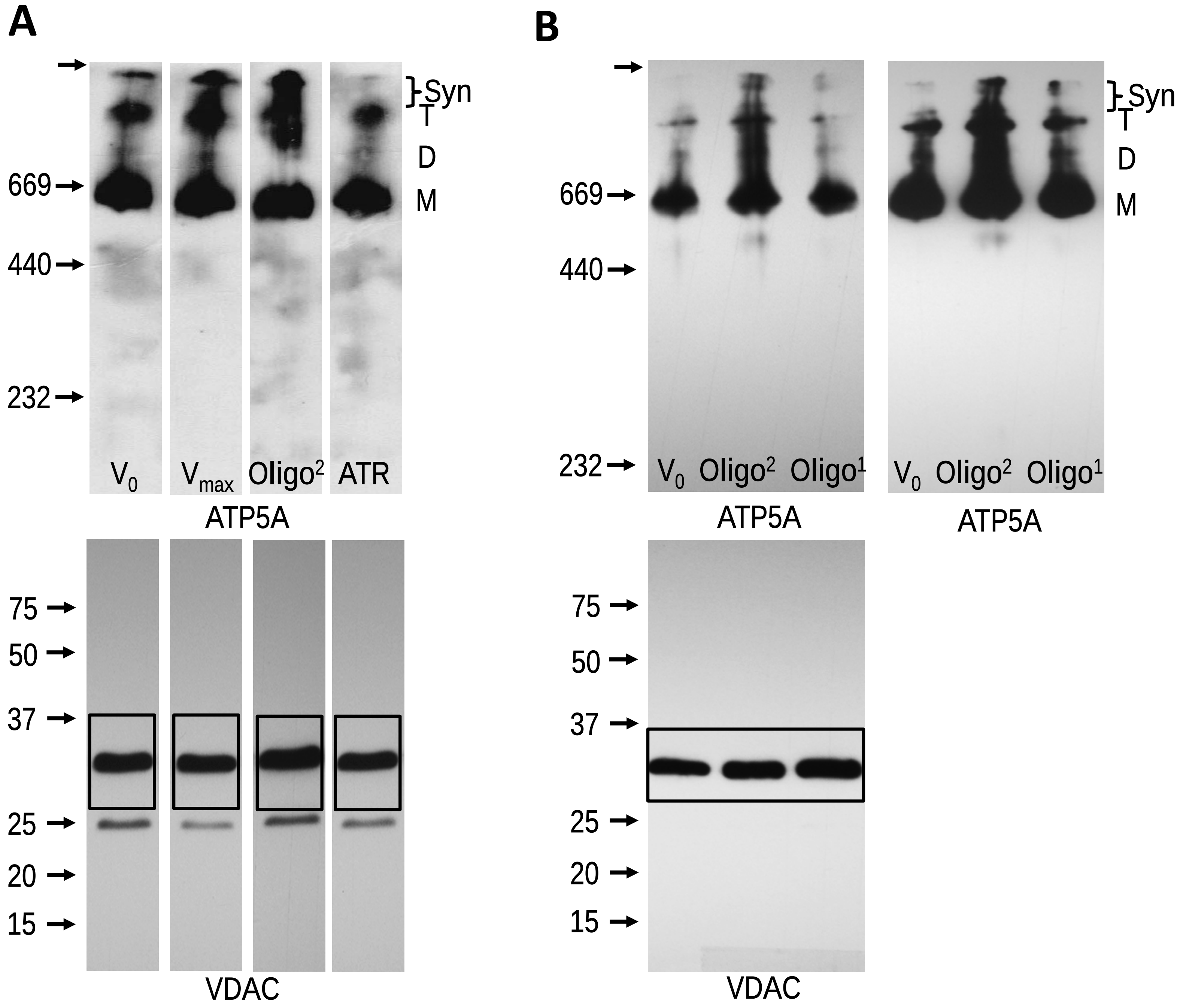

Supplement Figure 3 -Beutner

Supplement Figure 3: Full lanes of CN blots shown in Figure 2B and C. Respiration stimulates the formation of synthasomes. A: Synthasome (Syn) levels are highest during V_{max} (set as 100 %) compared to V_0 and ATR (n=6), while oligomycin ($Oligo^2$) had no significant effect (n=3). B: Addition of oligomycin (2 µg/ml) at V_{max} ($Oligo^2$) did not impact synthasome assembly but the addition of oligomycin before stimulation of OXPHOS ($Oligo^1$) prevented the assembly of synthasomes. This panel shows 2 different exposure times of the same blot. M, D and T refer to monomers, dimers and tetramers of ATP synthase. Below each CN blot the entire lanes or blots against VDAC are shown. Boxed areas indicate bands shown in Figure 2 B and C of the main manuscript. Positions of MW markers (in kDa) for CN or SDS IBs are presented to the left of IBs and the arrow at the top of CN blots indicates the bottom of the wells of the CN PAGE.

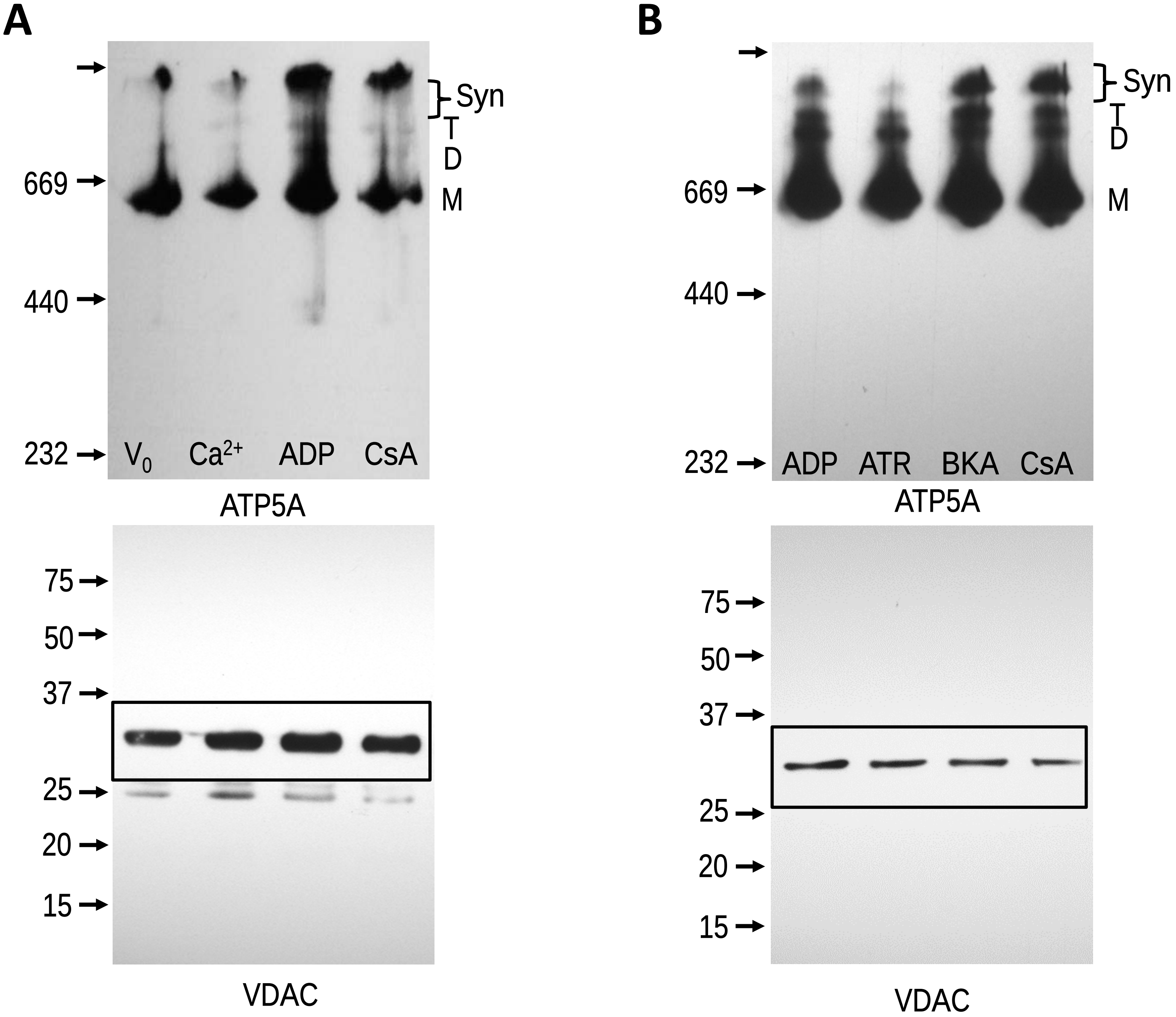

Supplement Figure 4-Beutner:

Supplement Figure 4: Full length lanes of CN blots presented in Figure 3B and C. Permeability transition leads to disassembly of synthasomes in WT hearts. A: This panel shows a representative CN blot, where Ca^{2+} keeps the level of synthasomes low while ADP or CsA preserve synthasomes. B: 0.5 mM ADP, 10 µM bongkrekic acid (BKA) and 200 nM CsA inhibit PT and preserve the synthasome, while 0.1 mM atractyloside (ATR) mediates PT and decreases synthasome levels (n=4). PT inhibitors or inducers were directly added to mitochondria in isotonic EGTA-free mannitol sucrose buffer and in the absence of substrates or Ca^{2+}. In both panels monomers (M), dimers (D), tetramers (T), and synthasomes (Syn) of the ATP synthase were labeled using anti-ATP5A. Positions of MW markers are indicated on the left and arrow at the top of CN blots indicates the bottom of the well. Below each CN blot the entire lanes of blots against VDAC are shown. Boxed areas indicate bands shown in Figure 3B and C of the main manuscript.

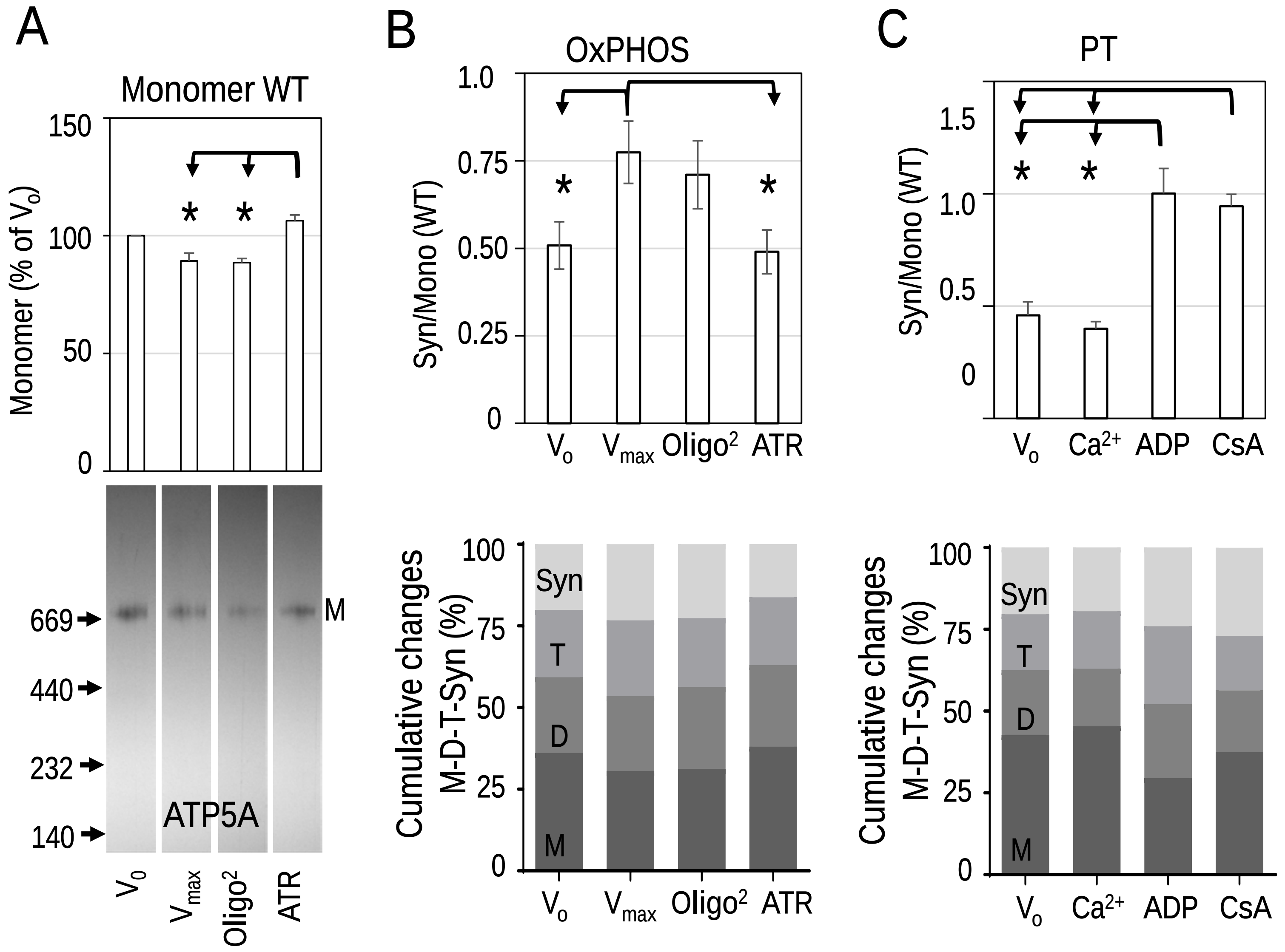

Supplement Figure 5-Beutner

Supplement Figure 5: Detection of synthasomes, tetramers, dimers and monomers in conditions representing OXPHOS (A, B) and PT (C) in wild type (WT) hearts. A: The top panel shows the relative levels of ATP synthase monomers is increased after inhibiting V_{max} with 0.1 mM ATR ($p \leq 0.01$). Fewer monomers were detected at V_{max} and when oligomycin (2µg/ml) were added at V_{max} ($oligo^2$) compared to ATR. The bottom panel shows a representative CN blot of the monomer of ATP synthase – visualized by ATP5A- after a very short exposure time. Please note that this exposure time is too short to visualize dimers, tetramers or synthasomes. B: The signal intensity of synthasomes (Syn), tetramers (T), dimers (D), and monomers (M) was determined with Image J. Top: The ratio of synthasomes to monomers indicates a transition to high molecular weight synthasomes during V_{max}, while the level of dimers and tetramers appears to be unchanged (Bottom). C: The ratio of synthasomes to monomers remains high if PT is prevented by ADP or CsA ($p \leq 0.01$). Conditions are as stated in Figure 3 (n=3).

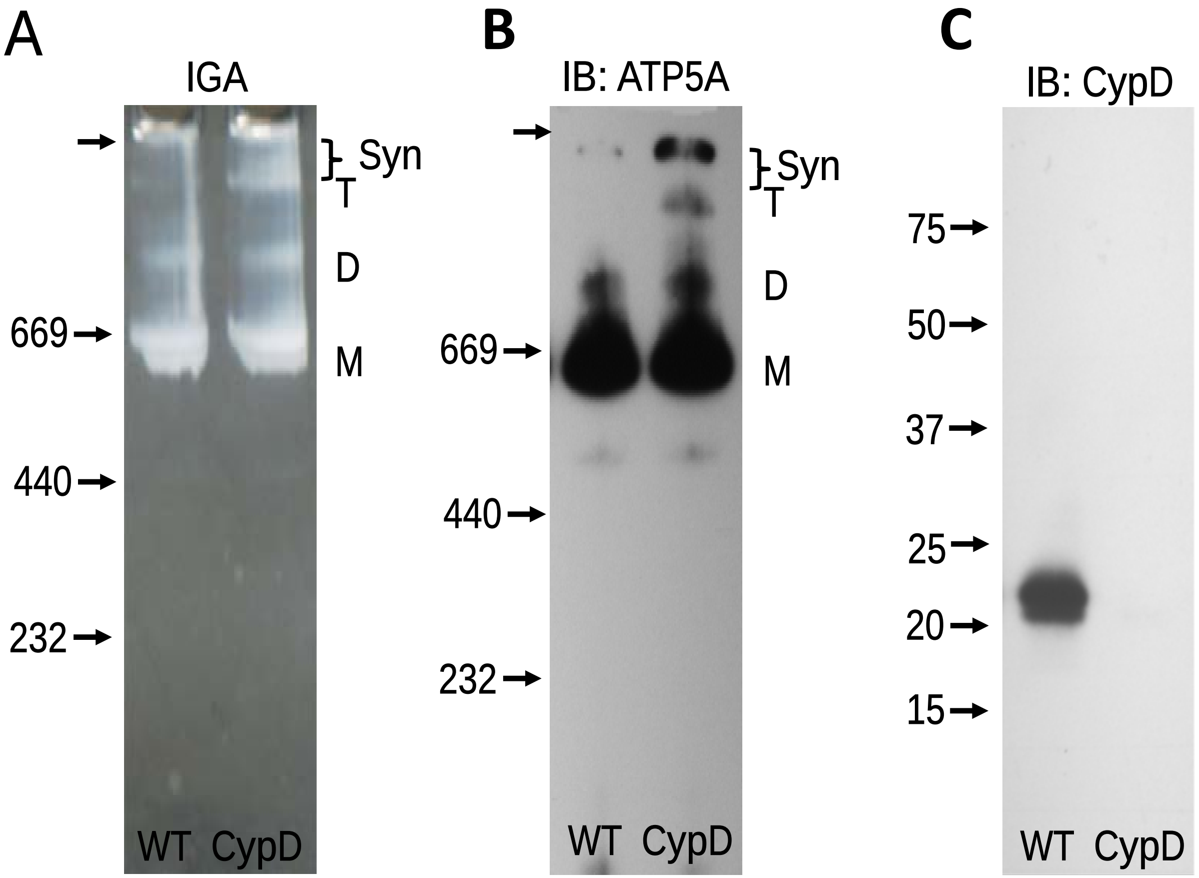

Supplement Figure 6-Beutner

Supplement Figure 6: Full CN blot and IGA as shown in Figure 4A. Synthasome levels are higher but less dynamic in hearts from CypD KO mice. A: ATP synthase in-gel assay (IGA) and B: immunoblotting (IB, for ATP5A) and after CN PAGE demonstrated more synthasomes (Syn) in CypD KO hearts compared to WT hearts. Note that the ATP synthase IGA results a white reaction product, so the shading is correct. In A and B, the arrow at the top of CN blots indicates the bottom of the well. C: No CypD was detectable by IB in heart mitochondria from CypD KO mice, while it was detected in WT hearts (SDS electrophoresis, 20 μg protein per lane).

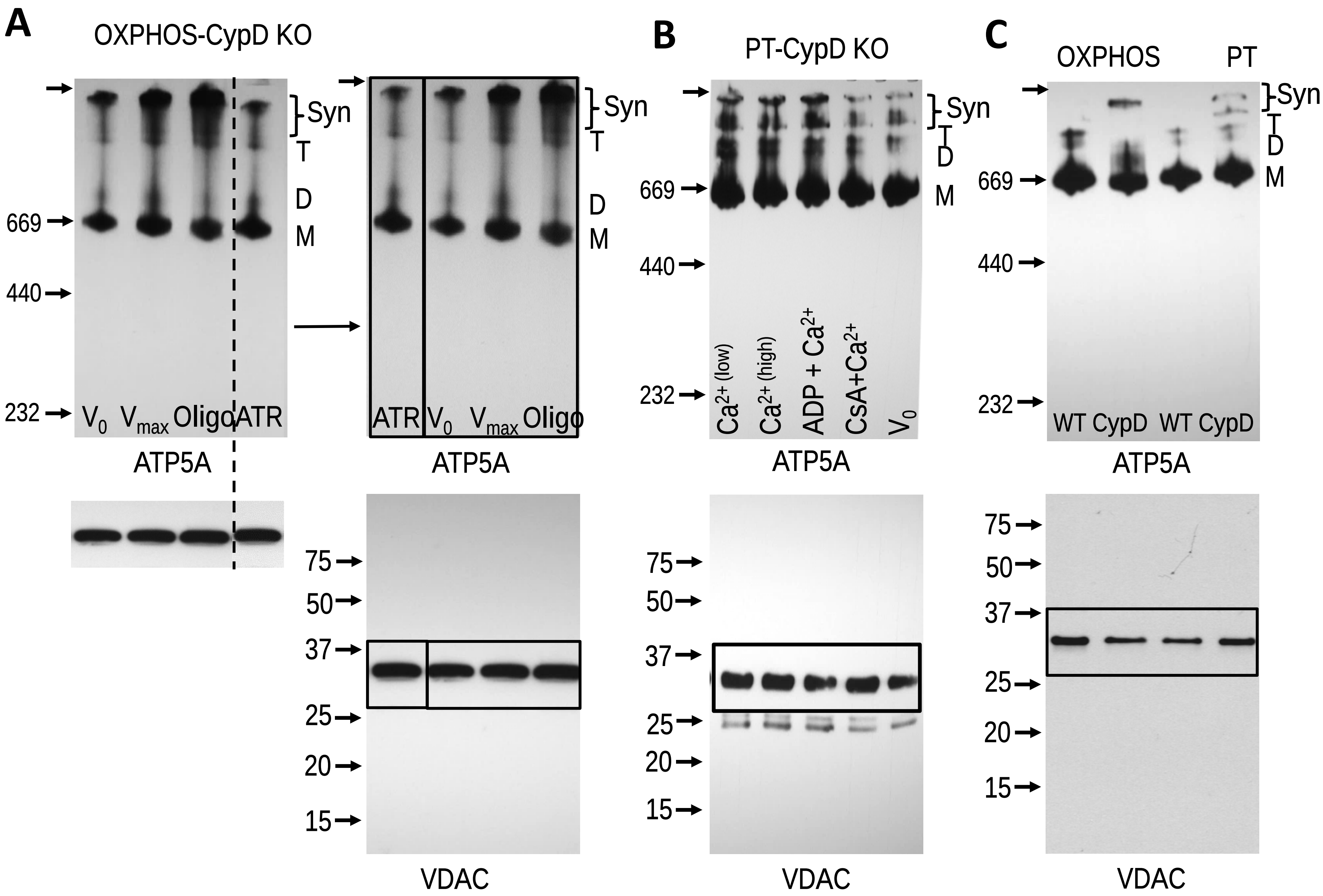

Supplement Figure 7-Beutner

Supplement Figure 7: Full length blots of Figure 4B, C and D. Synthasome levels are higher but less dynamic in hearts from CypD KO mice. A and B: No significant changes were observed in synthasome levels in CypD KO mitochondria during OXPHOS (B, n=3) and Ca^{2+}-induced PT (C, n=4). In A the left panel shows the blot as presented in the main manuscript, while the right panel shows the original arrangement of the lanes of the same blot. The experimental conditions are indicated. (Abbreviations and concentrations: V_0: 3 mM malate/5 mM glutamate, V_{max}: 1 mM ADP, ATR: 0.1 mM atractyloside, Oligo: 2 µg/ml oligomycin (added at V_{max}), ADP + Ca^{2+}: 0.5 mM ADP + 1 mM Ca^{2+}, CsA + Ca^{2+}: 200 nM cyclosporin A and 1 mM Ca^{2+}, $Ca^{2+ (low)}$: 60 µM, $Ca^{2+ (high)}$: 1 mM). No groups were significantly different by ANOVA. C: WT and CypD KO mitochondria run on the same gel featuring the experimental conditions in A (+ Mg^{2+}) and B (no Mg^{2+}) and show patterns similar to Figure 4B and C of the main manuscript, respectively. M, D, and T refer to monomers, dimers and tetramers of the ATP synthase, respectively. Positions of MW markers (in kDa) for CN IBs are presented to the left of IBs. Arrow at the top of CN blots indicates the bottom of the well. Below each CN blot the entire lanes of blots against VDAC are shown. Boxed areas indicate bands shown in Figure 4B, C and D of the main manuscript.

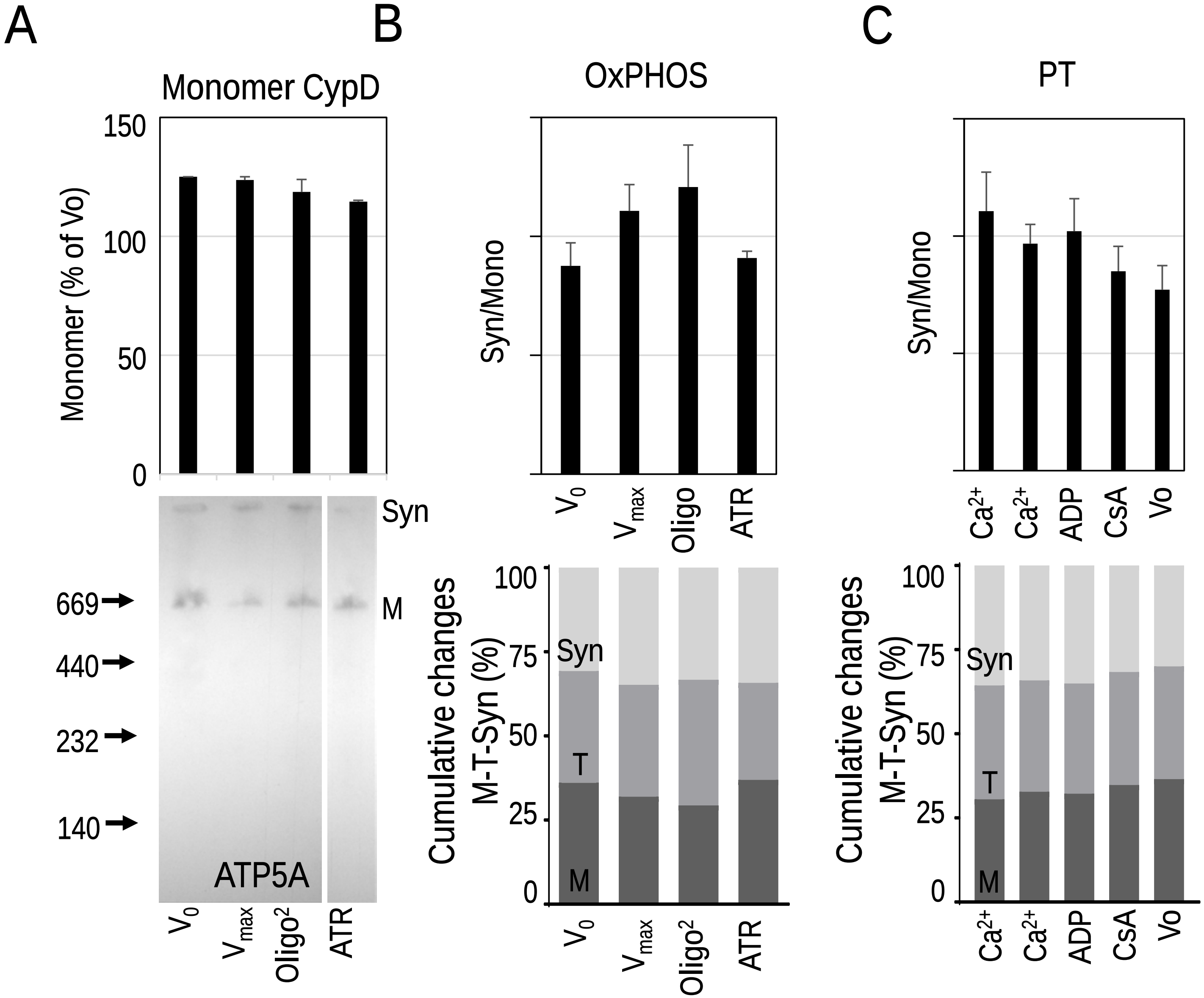

Supplement Figure 8-Beutner

Supplement Figure 8: Detection of synthasomes, tetramers, and monomers conditions representing OXPHOS (A, B) and PT (C) in mitochondria from CypD KO hearts. A: The top panel shows that the relative level of ATP synthase monomers remains unchanged independent of the experimental conditions. The bottom panel shows a representative CN blot of the monomer and synthasomes of ATP synthase – visualized by ATP5A- after a very short exposure time. Please note that this exposure time was not sufficient to visualize dimers or tetramers of the ATP synthase in CypD KO hearts, and dimers were inconsistently observed in immunoblots using CyPD null hearts. B and C: The signal intensity of synthasomes (Syn), tetramers (T), and monomers (M) was determined with Image J (n=5) and the ratio of synthasomes to monomers (Top) or synthasomes and tetramers to monomers (Bottom) did not significantly change in any condition using conditions representing OXPHOS or PTP (n=3). Conditions are as stated in Figure 4 (n=3).

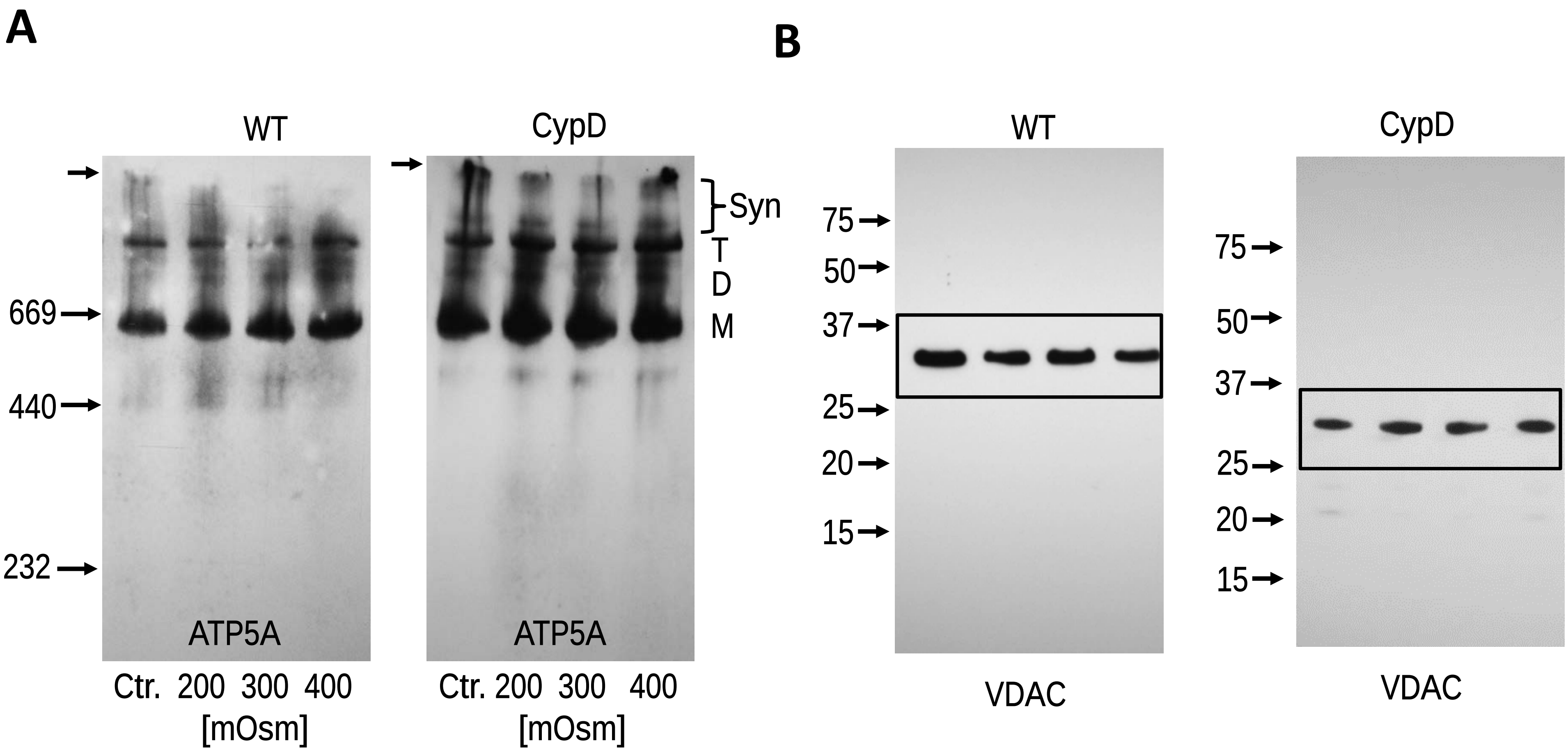

Supplement Figure 9-Beutner

Supplement Figure 9: Full length CN and SDS blots as shown in Figure 5. Formation of synthasomes during V_{max} is not due to changes of the mitochondrial matrix osmolarity. Formation of synthasomes during V_{max} is not due to changes of the mitochondrial matrix osmolarity. A: Isolated mitochondria from WT and CypD KO hearts (250 µg) were exposed for 15 minutes to a buffer (0.5 ml) where the concentration of mannitol and sucrose were adjusted so that the final osmolarity was normal (300 mOsm), low (200 mOsm), and high (400 mOsm)[24] or in EGTA-free mannitol/sucrose buffer (Ctr). M, D, T, and Syn refer to monomers, dimers, tetramers, and synthasomes of ATP synthase, respectively. Positions of MW markers (in kDa) for CN IBs are presented to the left of IBs. The right 2 panels show VDAC detection to confirm equal protein loading and the boxed areas are shown in Figure 5A of the main manuscript. Arrow at the top of CN blots indicates the bottom of the well.

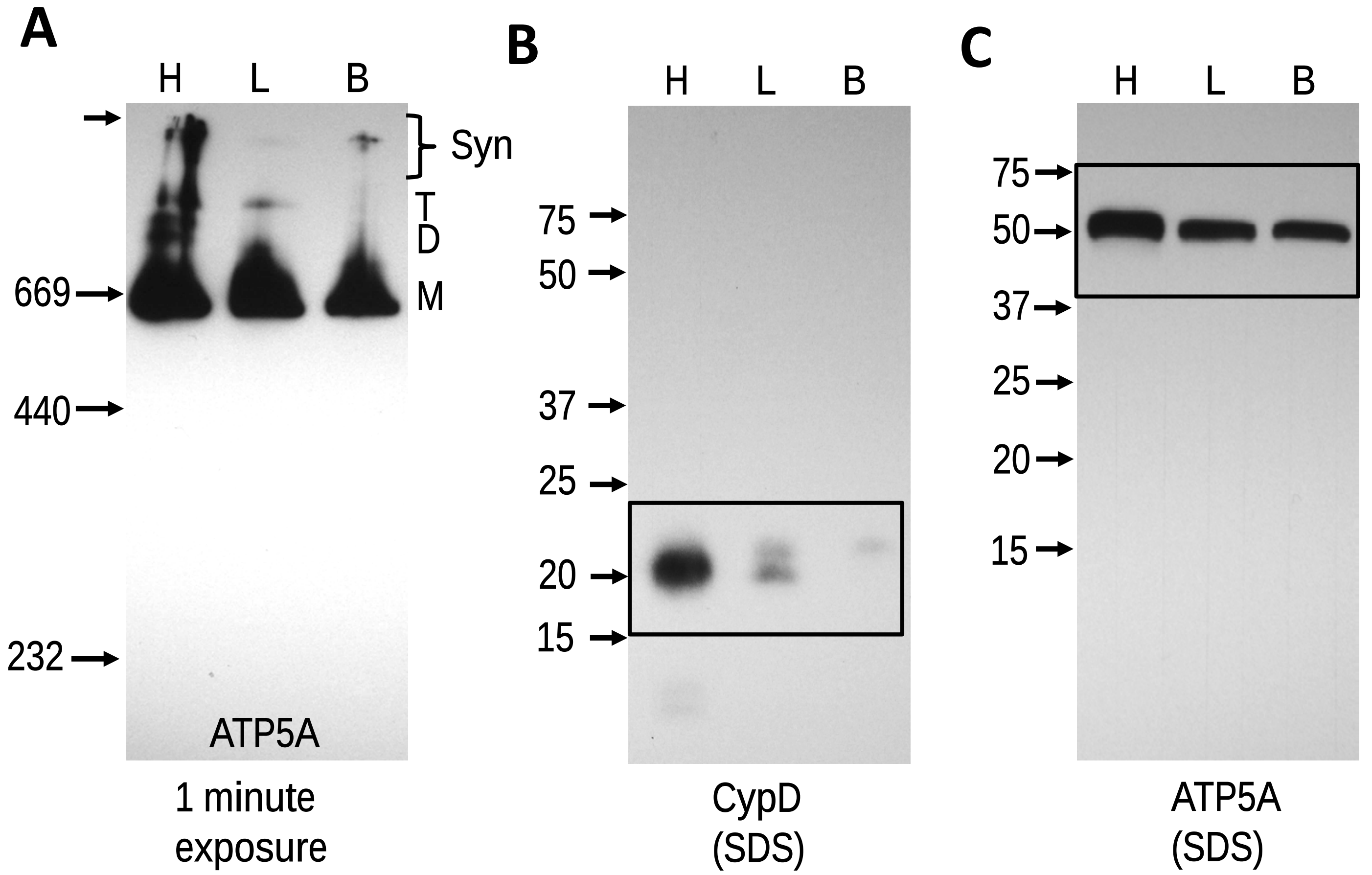

Supplement Figure 10-Beutner

Supplement Figure 10: Full length blots of blots as shown in Figure 6: CypD activity correlates with synthasome levels in heart, liver and brain mitochondria. A: CN PAGE of heart (H), liver (L) and brain (B) mitochondria show distinct patterns of ATP synthase assembly (left, M, D, T, and Syn refer to monomers, dimers, tetramers, and synthasomes respectively). Arrow at the top of CN blots indicates the bottom of the well. B: Total expression of CypD is lower in the brain compared to liver and heart in denaturing IBs. C: Full length blot as presented in 6E. Boxed areas in B and C are presented in Figure 6C and E of the main manuscript.

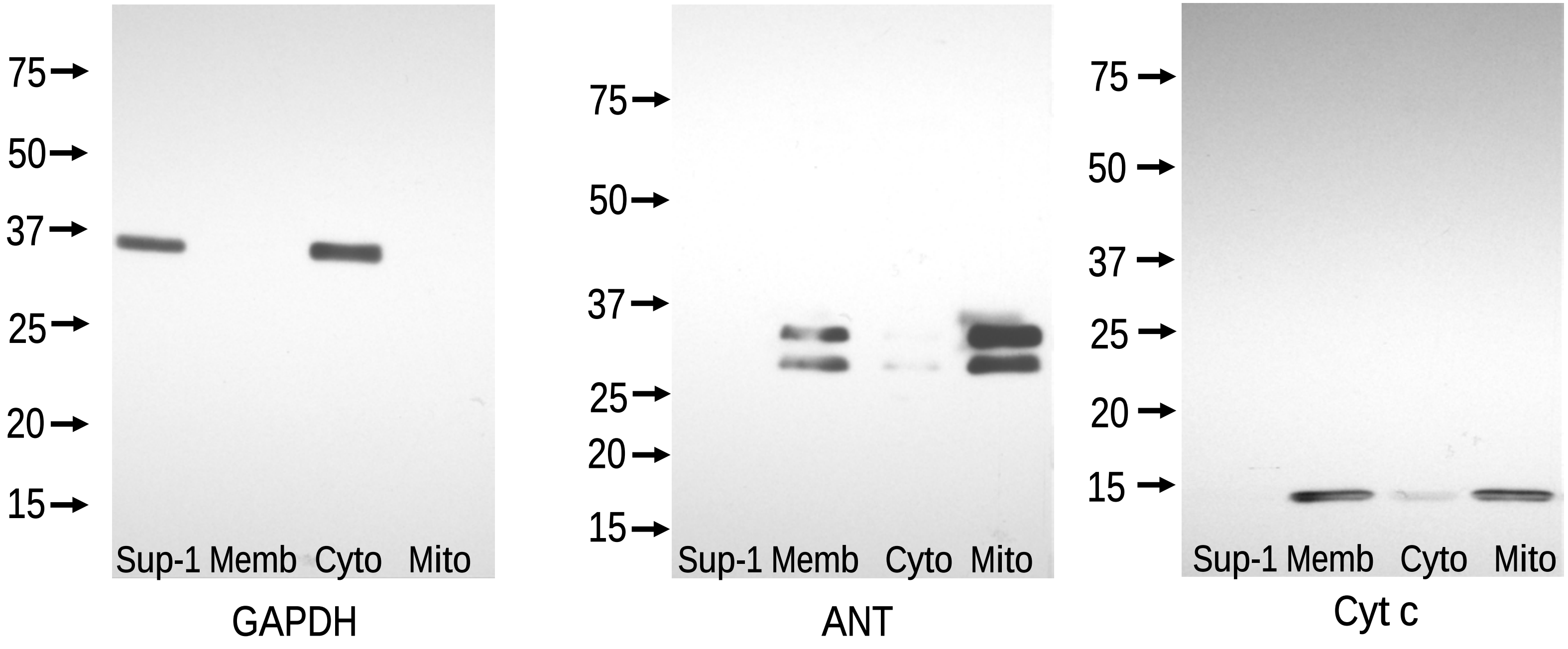

Supplement Figure 11-Beutner

Supplement Figure 11: Isolation of mitochondria from mouse hearts. The supernatant obtained after mincing the heart tissue and digesting it with a protease (Sup-1) contained glyceraldehyde dehydrogenase (GAPDH), but not the mitochondrial proteins ANT and cytochrome c (Cytc). The post-mitochondrial supernatant contains the cytosolic (Cyto) protein GAPDH, but only very little ANT and cytochrome c (Cytc). The mitochondria (Mito) enriched sediment is devoid of glyceraldehyde dehydrogenase (GAPDH), but was highly enriched in ANT and cytochrome c (Cytc). 20 µg protein were separated by SDS electrophoresis and, after transfer onto nitrocellulose membrane, labeled for the indicated proteins.
